# Drug–target interaction predictions with multi-view similarity network fusion strategy and deep interactive attention mechanism

**DOI:** 10.1093/bioinformatics/btae346

**Published:** 2024-06-05

**Authors:** Wei Song, Lewen Xu, Chenguang Han, Zhen Tian, Quan Zou

**Affiliations:** School of Computer and Artificial Intelligence, Zhengzhou University, Zhengzhou 450000, China; School of Computer and Artificial Intelligence, Zhengzhou University, Zhengzhou 450000, China; School of Computer and Artificial Intelligence, Zhengzhou University, Zhengzhou 450000, China; School of Computer and Artificial Intelligence, Zhengzhou University, Zhengzhou 450000, China; Yangtze Delta Region Institute (Quzhou), University of Electronic Science and Technology of China, Quzhou 324000, China; Yangtze Delta Region Institute (Quzhou), University of Electronic Science and Technology of China, Quzhou 324000, China

## Abstract

**Motivation:**

Accurately identifying the drug–target interactions (DTIs) is one of the crucial steps in the drug discovery and drug repositioning process. Currently, many computational-based models have already been proposed for DTI prediction and achieved some significant improvement. However, these approaches pay little attention to fuse the multi-view similarity networks related to drugs and targets in an appropriate way. Besides, how to fully incorporate the known interaction relationships to accurately represent drugs and targets is not well investigated. Therefore, there is still a need to improve the accuracy of DTI prediction models.

**Results:**

In this study, we propose a novel approach that employs Multi-view similarity network fusion strategy and deep Interactive attention mechanism to predict Drug–Target Interactions (MIDTI). First, MIDTI constructs multi-view similarity networks of drugs and targets with their diverse information and integrates these similarity networks effectively in an unsupervised manner. Then, MIDTI obtains the embeddings of drugs and targets from multi-type networks simultaneously. After that, MIDTI adopts the deep interactive attention mechanism to further learn their discriminative embeddings comprehensively with the known DTI relationships. Finally, we feed the learned representations of drugs and targets to the multilayer perceptron model and predict the underlying interactions. Extensive results indicate that MIDTI significantly outperforms other baseline methods on the DTI prediction task. The results of the ablation experiments also confirm the effectiveness of the attention mechanism in the multi-view similarity network fusion strategy and the deep interactive attention mechanism.

**Availability and implementation:**

https://github.com/XuLew/MIDTI.

## 1 Introduction

The prediction of drug–target interactions (DTIs) holds a critical position in the process of drug development and repurposing (Yıldırım *et al.* 2007). Researchers estimate that the development of one new drug to be approved by the traditional wet experimental approach for clinical use will cost over $1 billion and take 10–15 years (Hinkson *et al.* 2020). Therefore, in pursuit of accelerating the drug and target screening process, researchers have resorted to computational-based approaches to assist the rapid design and development of drugs (Lin *et al.* 2020). The essential step for these computational models is the prediction of underlying DTIs, aiming to identify novel targets for existing drugs.

Computational-based DTI prediction approaches (DTIs), could be divided into three categories: structure-based approaches, ligand-based approaches, and machine learning-based approaches (Ding *et al.* 2022). Structure-based and ligand-based methods are two types of traditional computation-based prediction approaches. Specifically, structure-based methods usually consider the structures of both drug molecules and their targets, as well as the binding sites (Shaikh *et al.* 2016). However, the known structures of some drug targets such as membrane proteins are still limited (Bagherian *et al.* 2021). Ligand-based approaches always utilize existing active small molecule structures to establish pharmacophore models or quantitative structure-activity relationships (Keiser *et al.* 2007). These methods usually need a large number of known binding ligands for interested targets and they are always ineffective when only a few ligands are known to bind with their targets (Tian *et al.* 2022).

Currently, many machine learning-based approaches have been widely proposed, which usually exploit the chemical structure of drugs and the genomic sequences of targets to extract their significant features efficiently. These methods always treat the DTI prediction problem as a binary classification task where they extract potential representations of drugs and targets, and take the concatenated embeddings of drug–target pairs as inputs for classification separately (Lee *et al.* 2019, Nguyen *et al.* 2021, Quan *et al.* 2019, Shin *et al.* 2019). For example, DeepConv-DTI learned the target protein features based on the convolution neural networks (CNN) model and the drug features from Extended Connectivity Fingerprint (ECFP) through the fully connected layer to predict DTIs. Nevertheless, the fully connected layer cannot capture potential relationships among distant atoms in raw molecule sequences (Lee *et al.* 2019). Meanwhile, the rapid development of graph neural networks (GNNs) has extended the application of machine learning to the graph domain, and related methods have also been applied for feature extraction. For example, GraphCPI (Quan *et al.* 2019) and GraphDTA (Nguyen *et al.* 2021) employed GNNs to capture the structural information of drugs and improve the predictive ability of DTIs. To comprehensively capture the relationship among atoms in the sequences, Shin *et al.* proposed a Transformer-based DTI model, which utilized multi-layered bidirectional transformer encoders to learn the high-dimensional structure of molecules from the simplified molecular input line entry system (SMILES) string (Shin *et al.* 2019). These methods above learned molecular representation only based on the molecular structure of drugs and targets themselves but ignored the interaction contributions between each DTI pair.

In addition to the chemical and genomic features, the relationships between biological entities (e.g. drugs, targets, diseases, and side effects) usually contain rich semantic information, which could offer a system-level understanding DTIs (Li *et al.* 2023). Thus, establishing meaningful networks that incorporate this heterogeneous biological information could contribute to the prediction of potential DTIs. For example, DITNet integrated diverse heterogeneous information and obtained the representations of nodes through the random walk with a restart (RWR) and diffusion component analysis (DCA) models (Luo *et al.* 2017). Based on DTINet, Peng *et al.* added a denoising-auto encoder-based feature selector and a CNN-based interaction predictor to improve the accuracy in DTI prediction (Peng *et al.* 2020). However, these approaches above usually extracted the features based on each network separately, which may be unable to leverage complex relationships from the heterogeneous networks consistently. Afterward, MVGCN employed a neighborhood information aggregation (NIA) layer designed for iteratively updating the embeddings of nodes from different views (Fu *et al.* 2022). EEG-DTI performed a message-passing strategy based on different types of edges in the heterogeneous networks (Peng *et al.* 2021). However, MVGCN and EEG-DTI considered different types of edges separately, which made it difficult to apply on large-scale networks with multiple types of data sources. It is also a challenge to integrate the representations of entities from multiple views in an appropriate manner.

Nowadays, various types of biological data related to drugs and targets are easily accessible, laying the foundation for multi-view similarity network construction. Since these networks may have varying rates of false positives and -negatives, a multi-view network fusion strategy should be raised for establishing a more robust biological network, which could accurately capture the underlying complex relationships. Meanwhile, previous studies only simply concatenate the representations of drugs and targets, which neglects the interactive contributions between the embeddings of drugs and targets. The attention mechanism in deep neural networks has shown an outstanding role in representation learning. Therefore, by means of the effective attention mechanism coupled with the known DTI information, we can obtain their discriminative representations in a feasible manner.

**Figure 1.**
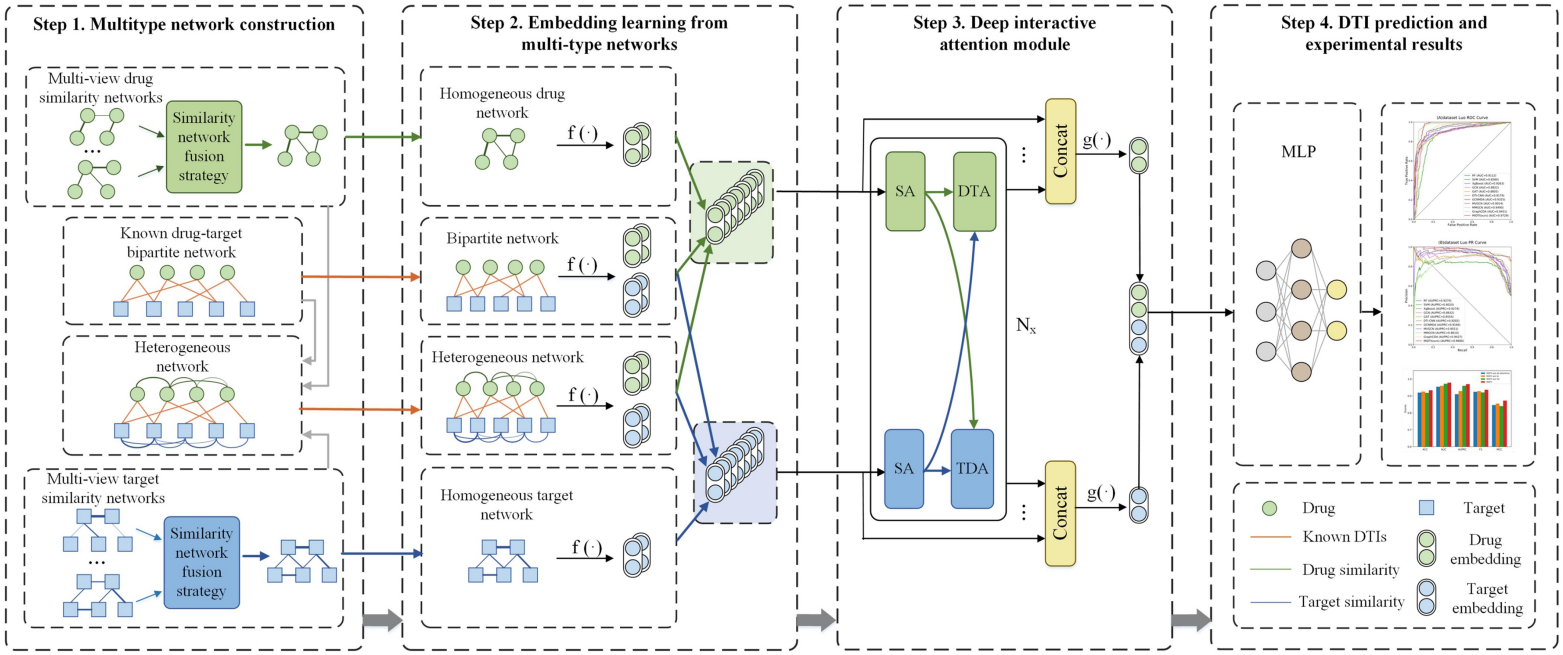
The overall framework of MIDTI. In Step 1, MIDTI constructs the integrated similarity networks of drugs and targets with their multisource information, as well as the drug–target bipartite network and the drug–target heterogeneous network. In Step 2, MIDTI learns the embeddings of drugs and targets from multiple networks respectively. In Step 3, MIDTI adopts the deep interactive attention mechanism to learn discriminative representations of drugs and targets. In Step 4, MIDTI predicts the potential DTIs with the MLP classifier.

Inspired by multi-view similarity network fusion strategy and deep interactive attention mechanism, here we propose a novel method called MIDTI to predict DTIs. The overall framework of MIDTI (see Fig. 1) mainly contains four steps. Firstly, MIDTI constructs different drug similarity networks based on drug-related association information and obtains an integrated drug similarity network with a multi-view similarity network fusion strategy. MIDTI also establishes an integrated target similarity network similarly. Secondly, MIDTI adopts the GCNs as the encoders to learn drug and target embeddings from the integrated drug similarity network, the integrated target similarity network, the drug–target bipartite network as well as the drug–target heterogeneous network respectively. Thirdly, MIDTI learns the discriminative embeddings based on the known DTI relationships with the deep interactive attention mechanism. Lastly, we feed the learned representations of drug–target pairs into the multilayer perceptron (MLP) to predict DTIs. Our main contributions can be summarized as follows:

- We put forward a novel multi-view similarity network fusion strategy, which could integrate different similarity networks in an unsupervised manner with the multi-view attention mechanism, as long as the nodes and sizes of these networks are consistent.
- MIDTI employs the deep interactive attention mechanism to learn the discriminative embeddings of drugs and targets with known DTI information.
- Extensive experimental results fully indicate that MIDTI is superior to other SOTA approaches in DTI prediction tasks.

## 2 Materials and methods

### 2.1 Data collection

In this study, DTIs were initially downloaded from Luo’s dataset (Luo *et al.* 2017). After processing, the experiment data mainly contains 12 015 nodes and 1 895 445 edges, which includes four types of nodes (drugs, targets, diseases, and side effects) and six types of edges (drug–protein interactions, drug–drug interactions, drug–disease associations, drug–side-effect associations, protein–disease associations, and protein–protein interactions). There are 1923 DTIs, related to 708 drugs and 1512 targets. Meanwhile, to comprehensively evaluate the performance of MIDTI, we also perform the comparison experiment on Yamanishi’s (Yamanishi *et al.* 2008) and Zheng’s dataset (Zheng *et al.* 2018). The description for these two datasets could be referred to the Supplementary Section S4.

### 2.2 Multitype network construction

As is shown in Step 1 in Fig. 1, we first construct different drug similarity networks and target similarity networks. Then MIDTI employs the similarity network fusion strategy to establish the integrated drug similarity network and the integrated target similarity network. Lastly, we construct the drug–target heterogeneous network.

#### 2.2.1 Similarity network construction for drugs and targets

MIDTI firstly establishes five similarity networks for drugs, based on (i) drug–drug interactions, (ii) drug–disease associations, (iii) drug–side-effect associations, (iv) drug–protein associations, and (v) drug–chemical structures. Meanwhile, MIDTI establishes four similarity networks for targets based on (i) protein–protein interactions, (ii) protein–disease associations, (iii) drug–protein associations, and (iv) genome sequences. The construction process for these similarity networks of drugs and targets has been displayed in Supplementary Section S1.

#### 2.2.2 Similarity network fusion strategy

**Figure 2.**
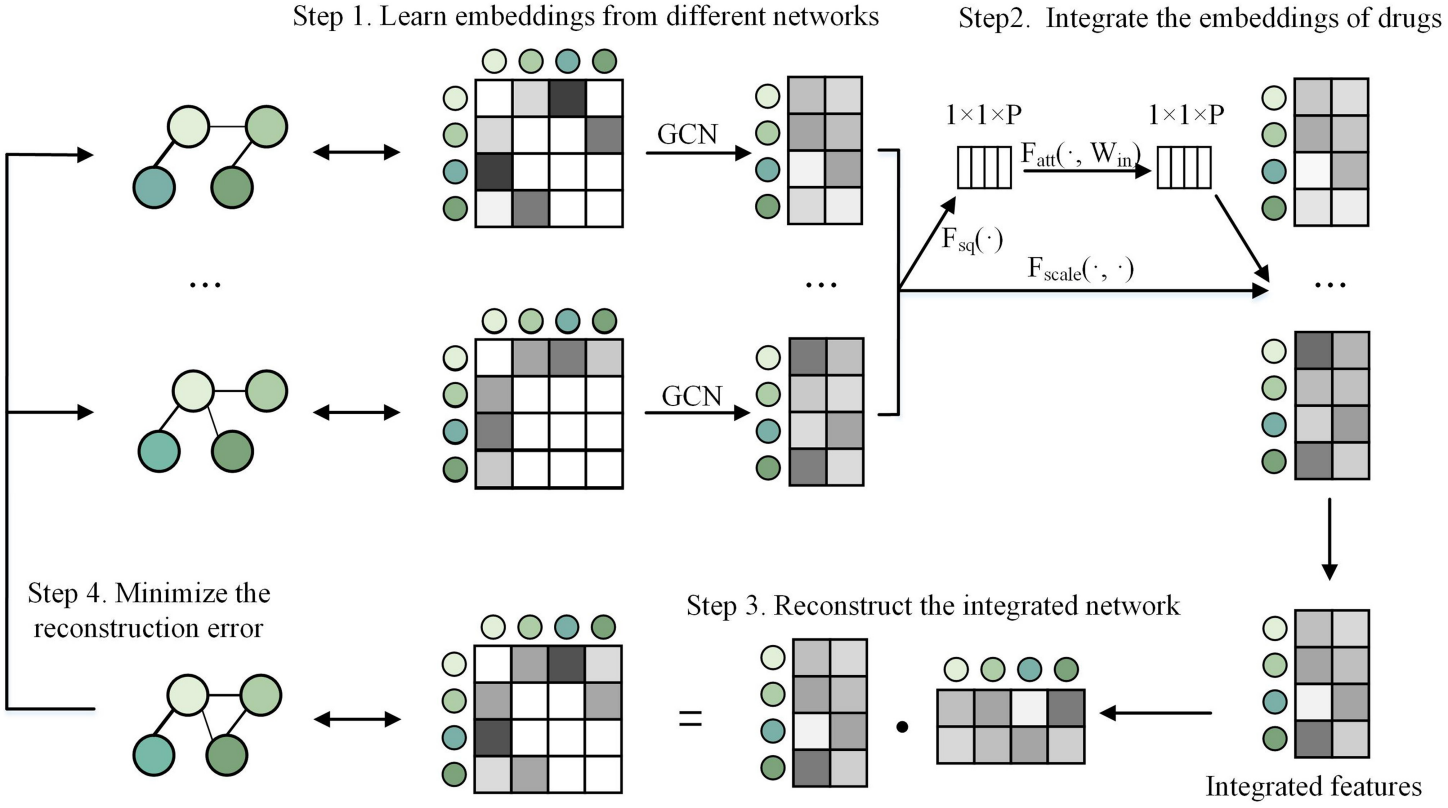
The four steps of multi-view drug similarity network fusion strategy. Step 1: Take different similarity networks of drugs as input and learn the embeddings of drugs from different networks. Step 2: Integrate the embeddings of drugs with the multi-view attention mechanism. Step 3: Reconstruct the integrated drug network through dot product operation on the integrated drug features. Step 4: Train MIDTI by minimizing reconstruction error between the reconstructed network and each original drug similarity network.

Inspired by BIONIC (Forster *et al.* 2022), MIDTI will integrate different similarity networks of drugs and targets with the similarity network fusion strategy (see Fig. 2). The integrated network could accurately reflect the topologies of the underlying original networks and capture functional information. Different from BIONIC, MIDTI adds a multi-view attention mechanism that adaptively learns the importance of features from different similarity networks. A detailed description of the similarity network fusion strategy has been presented in Supplementary Section S2.

In this way, MIDTI could establish the integrated drug similarity matrix $A_{\mathrm{homo}\_d}$ and the learned drug feature representation *X_d_*. Besides, MIDTI could also establish the integrated target similarity matrix and target feature matrix represented as $A_{\mathrm{homo}\_t}$ and *X_t_*.

#### 2.2.3 Drug–target heterogeneous network construction

MIDTI establishes a drug–target heterogeneous network named *N_hete_* based on the integrated drug similarity network, integrated target similarity network and drug–target bipartite network.

### 2.3 Embedding learning from multitype networks

In this section, MIDTI learns the embeddings of drugs and targets with GCNs from multi-type networks, which are integrated drug similarity network $N_{\mathrm{homo}\_d}$, integrated target similarity network $N_{\mathrm{homo}\_t}$, drug–target bipartite network *N_bi_* and drug–target heterogeneous network *N_hete_*, respectively. Meanwhile, their corresponding adjacency matrices are denoted as $A_{\mathrm{homo}\_d},\;A_{\mathrm{homo}\_t},\;A_{bi}$ and *A_hete_*.

Taking the $N_{\mathrm{homo}\_d}$ as an example, MIDTI adopts GCNs to learn embeddings of drugs and the output of (l+1) layer is denoted as:

(1) $$X_{\mathrm{homo}\_d}^{\left(l+1\right)}=\sigma \left({\tilde{D}}^{-\frac{1}{2}}{\tilde{A}}_{\mathrm{homo}\_d}{\tilde{D}}^{-\frac{1}{2}}X_{\mathrm{homo}\_d}^{\left(l\right)}W_{\mathrm{homo}\_d}^{\left(l\right)}\right)$$

where ${\tilde{A}}_{\mathrm{homo}\_d}=A_{\mathrm{homo}\_d}+I$ and *I* is the identity matrix with the same shape as $A_{\mathrm{homo}\_d},\;\tilde{D}$ is the degree matrix of $A_{\mathrm{homo}\_d}$. Besides, feature matrix $X_{\mathrm{homo}\_d}^{\left(0\right)}=X_{d}$.

Similarly, MIDTI learns the embeddings of drugs from *N_bi_* and *N_hete_* networks. The output at (l+1) layer is denoted as $X_{bi\_d}^{\left(l+1\right)}$ and $X_{\mathrm{hete}\_d}^{\left(l+1\right)}$, respectively.

Besides, MIDTI learns the embeddings of targets from $N_{\mathrm{homo}\_t}$, *N_bi_*, and *N_hete_* networks in a similar way. The output at (l+1) layer is denoted as $X_{\mathrm{homo}\_t}^{\left(l+1\right)},\;X_{bi\_t}^{\left(l+1\right)}$, and $X_{\mathrm{hete}\_t}^{\left(l+1\right)}$, respectively.

Given one drug, MIDTI learns its embeddings from each layer based on each network and stacks them together in a concatenated manner, which is formulated as:

(2) $$\begin{array}{cc} x_{d}= & \{x_{\mathrm{homo}\_d}^{\left(1\right)},x_{\mathrm{homo}\_d}^{\left(2\right)},\ldots ,x_{\mathrm{homo}\_d}^{\left(l\right)},x_{bi\_d}^{\left(1\right)},x_{bi\_d}^{\left(2\right)}\\ & ,\ldots ,x_{bi\_d}^{\left(l\right)},x_{\mathrm{hete}\_d}^{\left(1\right)},x_{\mathrm{hete}\_d}^{\left(2\right)},\ldots ,x_{\mathrm{hete}\_d}^{\left(l\right)}\} \end{array}$$

where $x_{d}\in R^{3l\times F_{m}}$. Likewise, the embedding of one target can be represented as:

(3) $$\begin{array}{cc} x_{t}= & \{x_{\mathrm{homo}\_t}^{\left(1\right)},x_{\mathrm{homo}\_t}^{\left(2\right)},\ldots ,x_{\mathrm{homo}\_t}^{\left(l\right)},x_{bi\_t}^{\left(1\right)},x_{bi\_t}^{\left(2\right)}\\ & ,\ldots ,x_{bi\_t}^{\left(l\right)},x_{\mathrm{hete}\_t}^{\left(1\right)},x_{\mathrm{hete}\_t}^{\left(2\right)},\ldots ,x_{\mathrm{hete}\_t}^{\left(l\right)}\} \end{array}$$

### 2.4 Deep interactive attention module

**Figure 3.**
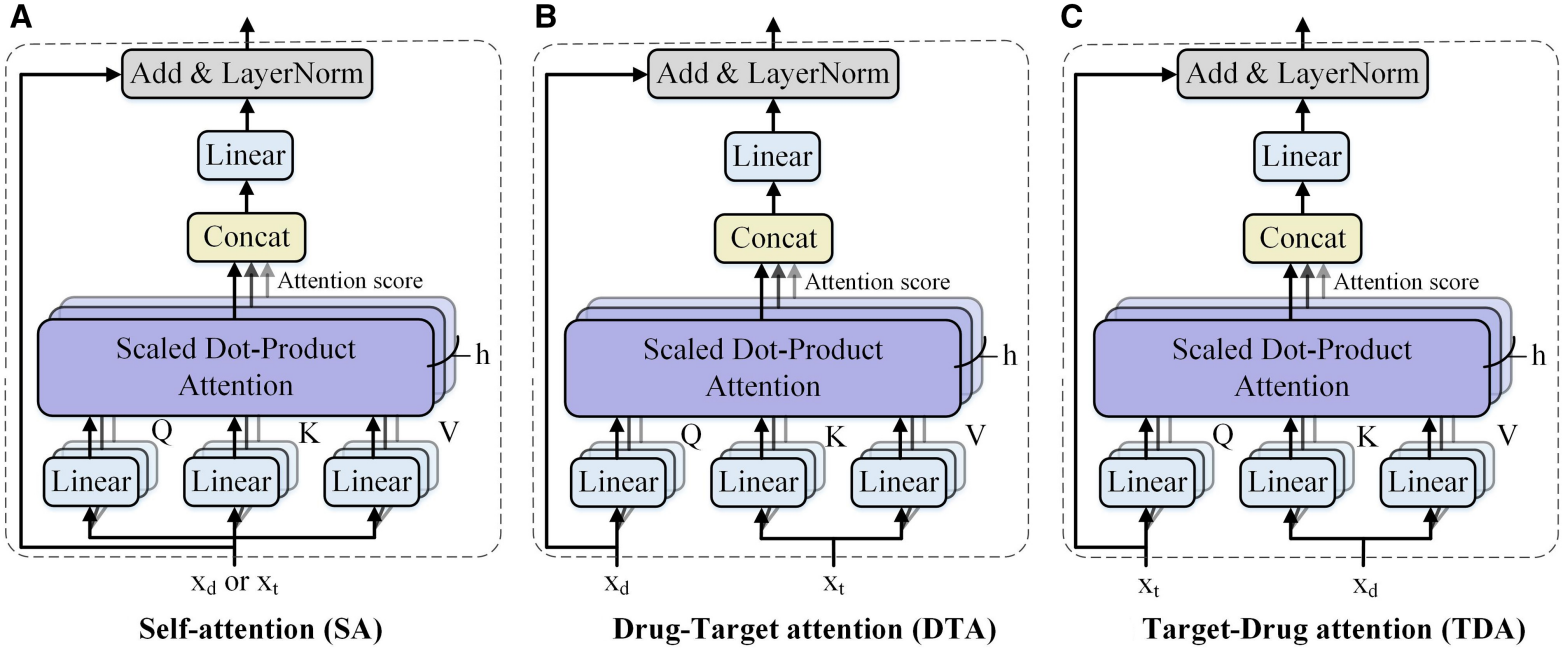
Three types of mechanism in deep interactive attention module. (A) SA mechanism, (B) DTA mechanism, (C) TDA mechanism.

In the deep interactive attention module, MIDTI employs the attention mechanism to further learn the discriminative representations of drugs and targets. Figure 3 demonstrates the three types of mechanisms in the deep interactive attention module, which are the self-attention (SA) mechanism, drug–target attention (DTA) mechanism, and target-drug attention (TDA) mechanism.

#### 2.4.1 Embedding learning with SA mechanism

Specifically, the SA mechanism takes *x_d_* (*x_t_*) as input and learns the embedding of drugs (targets), which is shown in Fig. 3A. The input of the scaled dot-product attention consists of three matrices: queries, keys and values, which are formulated as follows:

(4) $$Q=\mathrm{Linea}r_{Q}\left(x_{\mathrm{emb}}\right)$$

(5) $$K=\mathrm{Linea}r_{K}\left(x_{\mathrm{emb}}\right)$$

(6) $$V=\mathrm{Linea}r_{V}\left(x_{\mathrm{emb}}\right)$$

where $x_{\mathrm{emb}}=x_{d}$ when feeding drug embeddings and $x_{\mathrm{emb}}=x_{t}$ when feeding target embeddings.

Based on the three matrices, the attention score can be calculated by

(7) $$\mathrm{Attention}\left(Q,K,V\right)=\mathrm{softmax}\left(\frac{QK^{T}}{\sqrt{d}}\right)V$$

where $\sqrt{d}$ turns the attention matrix into the standard normal distribution.

To learn the embeddings of drugs and targets from different representation subspaces, the multi-head attention (MHA) mechanism is incorporated into the SA mechanism. The embedding of *x_d_* obtained with the MHA mechanism is represented as $x_{\left(d,\mathrm{mha}\right)}\in R^{3l\times F_{m}}$, which would be fed into the feed-forward layer and dropout layer.

The residual connection and layer normalization are incorporated to further improve the robustness of MIDTI. Finally, the embedding of drug *x_d_* with SA mechanism is presented as:

(8) $$x_{d}=\mathrm{Layernorm}\left(x_{\left(d,\mathrm{mha}\right)}+\mathrm{Dropout}\left(FL\left(x_{\left(d,\mathrm{mha}\right)}\right)\right)\right)$$

where FL(·) denotes the feed-forward layer, Dropout(·) denotes the dropout layer, and Layernorm(·) denotes the operation of layer normalization.

#### 2.4.2 Embedding learning with DTA and TDA mechanism

DTA mechanism is designed to estimate the contributions of different parts of a target to the drug (Fig. 3B). To be specific, DTA receives two inputs, which are *x_d_* and *x_t_*. Queries are computed by *x_d_*, and keys and values are obtained by *x_t_*. Hence the embeddings of targets guide the drug representations learning in DTA according to the attention scores.

Analogously, TDA also receives *x_d_* and *x_t_* but measures the effect of drug embedding on learning the target embedding (Fig. 3C). Keys and values are obtained by *x_d_* and queries are computed by *x_t_*.

#### 2.4.3 Embedding learning with deep interactive attention mechanism

**Figure 4.**
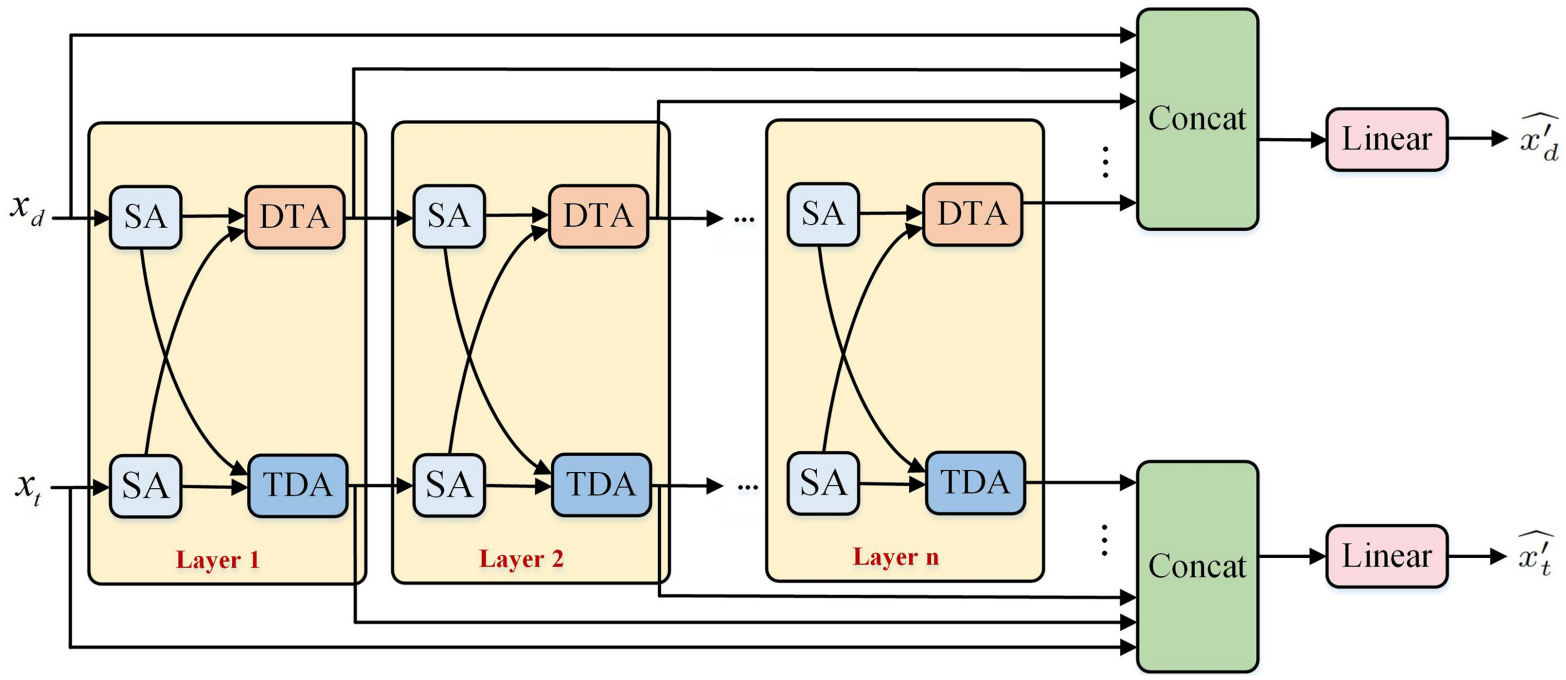
The deep interactive attention mechanism based on a cascade of interactive attention layers. Each interactive attention layer contains the corresponding SA, DTA, and TDA mechanisms respectively.

We put forward the interactive attention layer which contains SA, DTA, and TDA mechanisms (see Fig. 4). The embeddings of drugs and targets are initially separately fed into the SA layer, and then the TDA mechanism updates the target features with the help of the contributions of drug embeddings, while the DTA mechanism updates the embedding of drugs with the help of target embeddings.

After the operation on multiple interactive attention layers, we can fully learn the embeddings of drugs and targets. The result of (n+1)th interactive attention layer is formulated as:

(9) $$x\prime_{d}^{\left(n+1\right)}=\mathrm{DTA}\left(SA\left(x\prime_{d}^{\left(n\right)}\right),SA\left(x\prime_{t}^{\left(n\right)}\right)\right)$$

(10) $$x\prime_{t}^{\left(n+1\right)}=\mathrm{TDA}\left(SA\left(x\prime_{t}^{\left(n\right)}\right),SA\left(x\prime_{d}^{\left(n\right)}\right)\right)$$

where $x\prime_{d}^{\left(n\right)},\;x\prime_{t}^{\left(n\right)}$ are the representations of drug and target through *n*th interactive attention layers, $x\prime_{d}^{\left(0\right)}=x_{d},\;x\prime_{t}^{\left(0\right)}=x_{t},\;SA\left(\cdot \right)$ denotes the operations of the SA mechanism, DTA(·) and TDA(·) denotes the operations of DTA and TDA mechanism respectively.

MIDTI is designed to concatenate the outputs of the 0-st to *n*th interactive attention layers. Then we transform them through a linear layer to restore the same dimension as the inputs, which is formulated as:

(11) $$x\prime_{d}=\left(x\prime_{d}^{\left(0\right)}∥x\prime_{d}^{\left(1\right)}∥x\prime_{d}^{\left(2\right)}\ldots ∥x\prime_{d}^{\left(n\right)}\right)W_{r}$$

(12) $$x\prime_{t}=\left(x\prime_{t}^{\left(1\right)}∥x\prime_{t}^{\left(1\right)}∥x\prime_{t}^{\left(2\right)}\ldots ∥x\prime_{t}^{\left(n\right)}\right)W_{s},$$

where ∥ denotes the concatenation operation, and $W_{r},W_{s}\in R^{(n+1)\cdot F_{m}\times F_{m}}$, and in this study $x\prime_{d},x\prime_{t}\in R^{3l\times F_{m}}$.

Finally, we average the above embeddings in the first dimension and obtain the final discriminative representations of drugs and targets, which are denoted as:

(13) $$\hat{x\prime_{d}}=\mathrm{mean}\left(x\prime_{d}\right)$$

(14) $$\hat{x\prime_{t}}=\mathrm{mean}\left(x\prime_{t}\right)$$

where $\hat{x\prime_{d}},\hat{x\prime_{t}}\in R^{1\times F_{m}}$.

### 2.5 DTI prediction

For the representation $\hat{x\prime_{d}}$ and $\hat{x\prime_{t}}\in R^{1\times F_{m}}$, we concatenate them to represent the embedding of the drug–target pair and feed it into the MLP decoder, which is formulated as:

(15) $$f=\hat{x\prime_{d}}∥\hat{x\prime_{t}}$$

(16) $$\hat{y}=\mathrm{Tanh}\left(W\cdot f+b\right)$$

where ∥ denotes the concatenation operation, $W\in R^{2F_{m}}$ is the weight matrix, b∈R is the bias, and *Tanh*(·) is the activation function. Besides, $\hat{y}$ is the predicted value of DTI, which denotes the interaction probability between the drug and the target.

MIDTI utilizes the cross-entropy loss as the objective function to train MIDTI, and the loss *L* is minimized as:

(17) $$L=-\frac{1}{N}\sum_{i}^{N}y_{i}\;\text{log}\;\left(\hat{y_{i}}\right)+\left(1-y_{i}\right)\;\text{log}\;\left(1-\hat{y_{i}}\right)$$

where $y_{i}\in \{0,1\}$ is the ground truth label, and $\hat{y_{i}}$ is the predicted label, and *N* is the number of training samples.

## 3 Results

### 3.1 Experimental setup and evaluation metrics

In this study, we conduct the experiments on three datasets, which are Luo’s (Luo *et al.* 2017), Yamanishi’s (Yamanishi *et al.* 2008), and Zheng’s dataset (Zheng *et al.* 2018). For each dataset, we consider all the known DTI pairs as positive samples, and the remained drug–target pairs as negative samples. We initially select positive samples at a ratio of 1:1, 1:5, and 1:10 with negative samples to form three experimental datasets, respectively. Besides, MIDTI adopts the 5-fold cross-validation strategy (Tian *et al.* 2022) to evaluate its performance.

In this study, we adopt Accuracy (ACC), Area Under the receiver operating Characteristic curve (AUC), Area Under the Precision-Recall curve (AUPR), F1 score and Matthews Correlation Coefficient (MCC) as the evaluation metrics. All comparison methods adopt the same 5-fold cross-validation (5-CV) as MIDTI, and the results shown here are the average values of the five-time experiments. The implementation details of the experiments are presented in Supplementary Section S3. The time and space complexity analysis for MIDTI is presented at Supplementary Section S10.

### 3.2 Comparison with other baseline methods

Ten competitive approaches are selected for comparison with MIDTI and we evaluate them with ACC, AUC and AUPR metrics. They are Random Forests (RF) (Pedregosa *et al.* 2011), Support-Vector Machine (SVM) (Chang and Lin 2011), eXtreme Gradient Boosting (XGBoost) (Chen and Guestrin 2016), GCN (Kipf and Welling 2016), Graph Attention Networks (GAT) (Veličković *et al.* 2017), DTI-CNN (Peng *et al.* 2020), GCNMDA (Long *et al.* 2020), MVGCN (Fu *et al.* 2022), MMGCN (Tang *et al.* 2021), GraphCDA (Dai *et al.* 2022), and DTINet (Luo *et al.* 2017). The description for these comparison approaches is presented in Supplementary Section S5.

**Figure 5.**
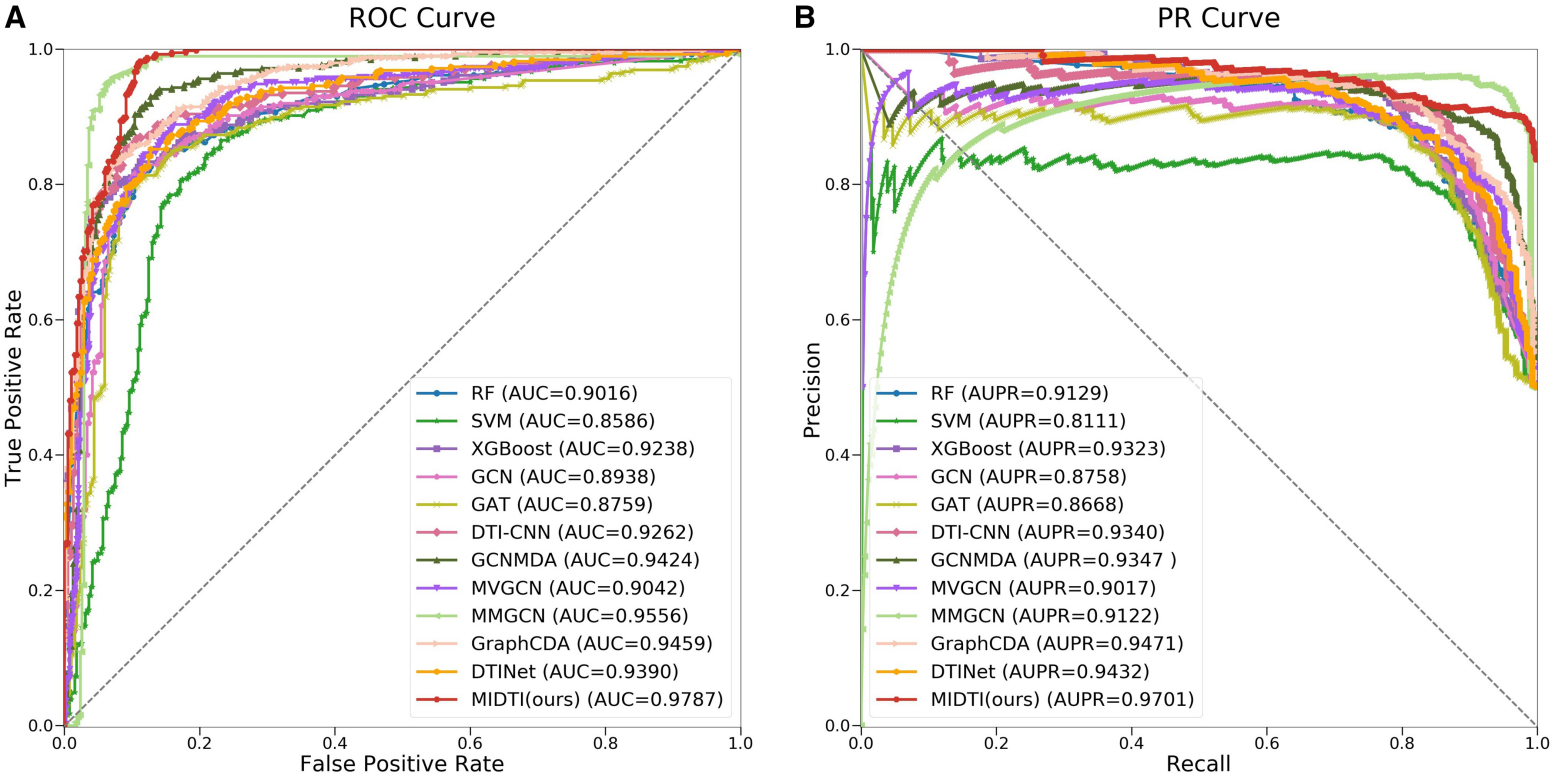
The results of MIDTI as well as other baseline approaches on AUC and AUPR metrics.

The comparison results are shown in Fig. 5 and Table 1, and Fig.5(A) and Fig.5(B) denote the ROC and PR curves respectively. MIDTI wins the best performance among all SOTA methods. Specifically, MIDTI gets the scores on ACC, AUC, and AUPR metrics are 0.9340, 0.9787, and 0.9701. MIDTI is 2.55%, 2.31%, and 2.30%, higher than the ACC of MMGCN, AUC of MMGCN and AUPR of GraphCDA, respectively. Preliminary experiments suggest that MIDTI is the most competitive drug–target association prediction method on this dataset. The comparison results under the 1:1 ratio on Yamanishi’s dataset are presented at Supplementary Section S6 in Supplementary Table S1. The comparison results under the 1:1 ratio on Zheng’s dataset are presented at Supplementary Section S6 in Supplementary Table S4.

**Table 1.** The performance of MIDTI as well as other baseline approaches for predicting DTI under different ratios on Luo’s dataset.^a^

Models	1:1			1:5			1:10		
	ACC	AUC	AUPR	ACC	AUC	AUPR	ACC	AUC	AUPR
RF (Pedregosa *et al.* 2011)	0.8409	0.9016	0.9129	0.9103	0.9093	0.7836	0.9438	0.9176	0.7156
SVM (Chang and Lin 2011)	0.7993	0.8586	0.8111	0.9074	0.8917	0.6962	0.9380	0.8871	0.6078
XGBoost (Chen and Guestrin 2016)	0.8573	0.9238	0.9323	0.7982	0.8586	0.8111	0.9550	0.9311	0.7864
GCN (Kipf and Welling 2016)	0.8393	0.8938	0.8758	0.9068	0.8895	0.7100	0.9299	0.8617	0.5817
GAT (Veličković *et al.* 2017)	0.8219	0.8759	0.8668	0.8710	0.8558	0.6339	0.9268	0.8525	0.5340
DTI-CNN (Peng *et al.* 2020)	0.8523	0.9262	0.9340	0.9269	0.9281	0.8286	0.9558	0.9319	0.7957
GCNMDA (Long *et al.* 2020)	0.8850	0.9424	0.9347	0.9044	0.9354	0.7520	0.9302	0.9423	0.6573
MVGCN (Fu *et al.* 2022)	0.8489	0.9042	0.9017	0.9132	0.9209	0.7777	0.9445	0.9163	0.6959
MMGCN (Tang *et al.* 2021)	0.9085	0.9556	0.9122	0.9403	0.9671	0.8038	**0.9582**	0.9715	0.7684
GraphCDA (Dai *et al.* 2022)	0.8796	0.9459	0.9471	0.9221	0.9484	0.8353	0.9377	0.9133	0.6435
DTINet (Luo *et al.* 2017)	0.8672	0.9390	0.9432	0.8983	0.9017	0.8511	0.9029	0.9003	0.7883
MIDTI (ours)	**0.9340**	**0.9787**	**0.9701**	**0.9413**	**0.9813**	**0.9075**	0.9539	**0.9794**	**0.8431**

a The best results are marked in bold and the second best is underlined.

### 3.3 Experimental results of MIDTI with different ratios between positive and negative samples

The different ratios of positive samples to negative samples can affect the performance of MIDTI and baseline methods. Hence, we conduct evaluation experiments under positive samples to negative sample ratios of 1:5 and 1:10 on Luo’s dataset.

For the results with the 1:5 ratio, MIDTI gets the first rank on ACC, AUC and AUPR, and their scores are 0.9413, 0.9813, and 0.9075, respectively. The scores of MMGCN (rank second) on ACC and AUC are 0.1% and 1.42% lower than those of MIDTI. DTINet is lower than MIDTI by 6.2% on AUPR metric. For the results with the 1:10 ratio, results show that MIDTI also performs best on AUC and AUPR metrics, which are 0.9794 and 0.8431. MMGCN achieves the highest score on ACC metric, which is 0.9582. The other results have already been presented in Table 1.

Besides, the comparison results under the 1:5 and 1:10 ratio on Yamanishi’s dataset are presented at Supplementary Section S6 in Supplementary Tables S2 and S3. The comparison results under 1:5 and 1:10 ratio on Zheng’s dataset are presented at Supplementary Section S6 in Supplementary Table S4.

### 3.4 Model ablation study

In order to verify the effectiveness of the essential modules in MIDTI, we conductthree sets of ablation experiments.

The first ablation experiment is to verify the effectiveness of each attention mechanism. MIDTI applies the attention mechanism at two stages: one is the multi-view attention (VA) mechanism to fuse multiple drug-similarity networks and target-similarity networks respectively, and the other is the deep interactive attention (IA) mechanism to learn the embedding of drugs and targets. Here, MIDTI splits VA and IA into four combinations, which are displayed in Table 2.

**Table 2.** The ablation experimental results on the view-attention mechanism and interactive attention mechanism for MIDTI.

VA	IA	ACC	AUC	AUPR	F1	MCC
✗	✗	0.9202	0.9547	0.9108	0.9247	0.8464
*✓*	✗	0.9259	0.9603	0.9291	0.9289	0.8550
✗	*✓*	0.9184	0.9723	0.9603	0.9212	0.8402
*✓*	*✓*	**0.9340**	**0.9787**	**0.9701**	**0.9370**	**0.8726**

The best results are in bold and the second best results are underlined.

The results in Table 2 demonstrate that MIDTI achieves the best performance in all five metrics. The values for ACC, AUC, AUPR, F1 and MCC are 0.9340, 0.9787, 0.9701, 0.9370, and 0.8726, respectively. And the performance of MIDTI w/o all attention is worst on AUC and AUPR since it does not employ any attention. Results indicate the deep interactive attention mechanism and the view-attention mechanism both play an essential role in improving the performance of MIDTI.

The second ablation experiment is to verify the effectiveness of the homogeneous similarity network, the bipartite network and the heterogeneous network of drugs and targets, which are denoted as *N_homo_*, *N_bi_*, *N_hete_*, respectively. Specifically, three networks are divided into seven different combinations. The results for each combination are displayed in Table 3.

**Table 3.** The ablation experimental results on the homogeneous similarity network, the bipartite network and the drug–target heterogeneous network for MIDTI.

N_*homo*_	N_*bi*_	N_*hete*_	ACC	AUC	AUPR	F1	MCC
*✓*	✗	✗	0.5637	0.7142	0.6681	0.6137	0.1870
✗	*✓*	✗	0.9192	0.9767	0.9583	0.9308	0.8701
✗	✗	*✓*	0.8648	0.9514	0.9380	0.8656	0.7412
*✓*	*✓*	✗	0.9229	0.9760	0.9615	0.9313	0.8714
*✓*	✗	*✓*	0.8692	0.9479	0.9332	0.8688	0.7399
✗	*✓*	*✓*	0.9273	0.9783	0.9688	0.9300	0.8715
*✓*	*✓*	*✓*	**0.9340**	**0.9787**	**0.9701**	**0.9370**	**0.8726**

The best results are in bold and the second best results are underlined.

The results demonstrate that MIDTI achieves the best performance in all five metrics, and the corresponding values are 0.9340, 0.9787, 0.9701, 0.9370, and 0.8726. The results in this set of experiments illustrate that all three similarity networks are essential in learning drug and target embeddings.

The third ablation experiment is to verify the effectiveness of the proposed similarity network fusion strategy. Here, we also select other two similar network fusion strategies, which are called MIDTI_ave and MIDTI_pro. For MIDTI_ave strategy, we measure the arithmetic average values from different networks as the integrated similarity values. For MIDTI_pro strategy, the integrated similarity value is formulated as $S=1-\prod_{i=1}^{n}S_{i}$, where *S_i_* denotes the similarity values from the *i*th similarity network. The corresponding results for each combination are displayed in Table 4. The results demonstrate that MIDTI achieved the best performance on Luo’s dataset and Zheng’s dataset, which could confirm the effectiveness of the proposed similarity network fusion strategy. The results on Yamanishi’s dataset are displayed in Supplementary Section S8.

**Table 4.** The evaluation results of MIDTI with different similarity network fusion strategy on Luo’s, and Zheng’s datasets.

Datasets	Strategy	ACC	AUC	AUPR
	MIDTI_ave	0.9078	0.9547	0.9336
Luo	MIDTI_pro	0.8961	0.9611	0.9501
	MIDTI	**0.9340**	**0.9787**	**0.9701**
	MIDTI_ave	0.8162	0.8789	0.8665
Zheng	MIDTI_pro	0.8048	0.8803	0.8714
	MIDTI	**0.8836**	**0.9546**	**0.9497**

The best results are in bold and the second best results are underlined.

### 3.5 Parameter analysis experiments

In this section, we discuss the sensitivity of several parameters of MIDTI. These parameters mainly include the embedding size, the learning rate of the optimizer, the number of interactive attention heads, the number of GCN layers, the number of interactive attention layers and the number of MLP layers. The corresponding experiment results are all evaluated with ACC, AUC, AUPR, F1, and MCC, respectively.

From the results shown in Supplementary Figs S1 and S2 in Supplementary Section S7, we can see that MIDTI adopts the embedding size, learning rate, the number of interactive heads, the number of GCN layers, the number of interactive attention layers, the number of MLP layers as 512, 0.1, 8, 3, 3, 3, respectively. A more detailed description of this group experiment is displayed in Supplementary Section S7.

### 3.6 Visualization and interpretation for the embeddings of drug–target pairs learned by MIDTI

**Figure 6.**
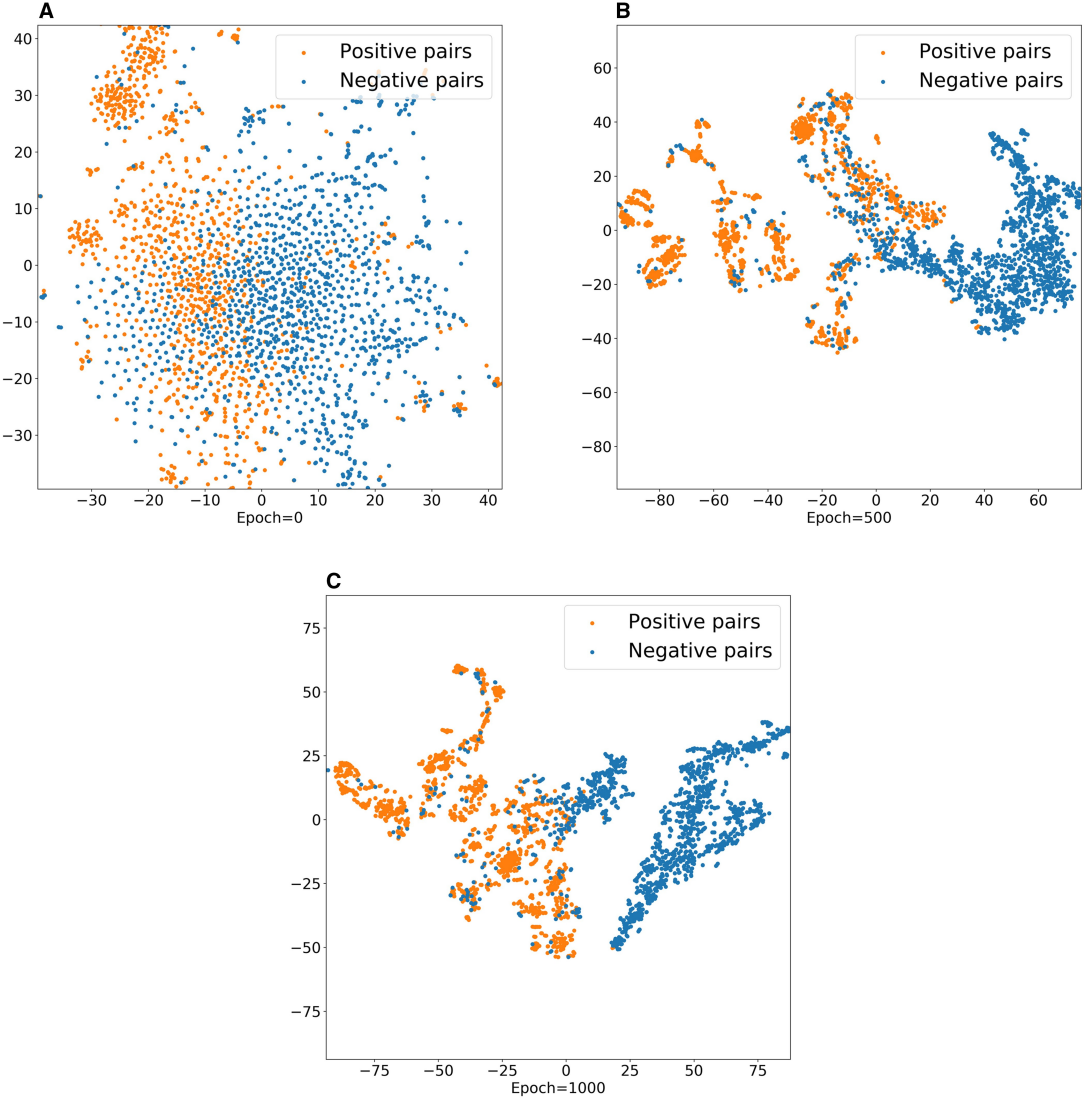
Visualization of the learned drug–target embeddings by MIDTI under different epochs.

To further demonstrate the ability of MIDTI in learning embeddings of drugs and targets, we conduct the visualization experiment. Specifically, with the learned embeddings of drugs and targets, we can generate embeddings for positive and negative drug–target pairs. All the embeddings of drug–target pairs are plotted into a 2D space using t-SNE tool (Van der Maaten and Hinton 2008). The visualization results are displayed in Fig. 6.

It can be seen that the positive pairs and the negative pairs are gradually distinguished with the epochs increasing (see Fig. 6(A, B, C)). The embeddings of positive pairs and the negative pairs are in chaos when the epoch number is 0. The embedding distribution is gradually clear with the epochs increase. Finally, the positive pairs (yellow points) and the negative pairs (blue points) are almost separated when the number of epochs equals 1000. This observation further confirms that the learned embeddings of drug–target pairs are discriminative and interpretable, which improves the accuracy of MIDTI in predicting DTIs.

### 3.7 Case study

In practice, discovering the interactions accurately for some common drugs and targets is another effective manner to verify the effectiveness of DTI prediction models (Tian *et al.* 2022). In this section, we mainly conduct two types of experiments, and the corresponding results have been presented in Supplementary Section S9. The predicted targets for selected drugs almost could be verified. The analysis indicates that MIDTI has the powerful ability to discover potential DTIs, which has essential implications for drug screening and drug repositioning.

## 4 Conclusion

In this study, we propose a novel method called MIDTI to predict DTIs. We conducted extensive experiments to evaluate the performance of MIDTI. The comparison results demonstrate that MIDTI achieves the best performance with different ratios. Besides, the ablation experiments and parameter sensitivity experiments are also performed to further confirm the effectiveness of MIDTI. Finally, the results of the case study are supported by different published databases.

Next, we could work on the following two aspects. Firstly, we can utilize other related data sources of drugs and targets for their embedding learning. Secondly, MIDTI can be applied to other link prediction problems, such as miRNA–disease association prediction.

## Supplementary Material

btae346_Supplementary_Data
