# Supplementary material for: Drug–target interaction predictions with multi-view similarity network fusion strategy and deep interactive attention mechanism
Source: Bioinformatics. 2024 Jun 5;40(6):btae346. doi: 10.1093/bioinformatics/btae346 (PMC11164831; doi:10.1093/bioinformatics/btae346)
Supplement: btae346_Supplementary_Data [file btae346_supplementary_data.pdf]

# Supplementary

## Section 1. MIDTI evaluates different similarities between drugs and targets with their multisource information.

Specifically, MIDTI adopts the Jaccard similarity coefficient (Niwattanakul *et al.*, 2013) to measure the similarity between drugs. We take the drug-disease associations as an example to display the similarity calculation process. Suppose there are two drugs named $D_1$ and $D_2$, and their corresponding associated diseases set denoted as $S_{D_1}$ and $S_{D_2}$, the similarity between $D_1$ and $D_2$ could be formulated as:

$$Sim(D_1, D_2) = \frac{|S_{D_1} \cap S_{D_2}|}{|S_{D_1} \cup S_{D_2}|} \tag{1}$$

In this way, we could measure the similarities for all the drug pairs and establish the corresponding drug-disease-based similarity network. MIDTI could construct the drug-drug interaction-based similarity network, drug-protein association-based similarity network, and drug-side-effect association-based similarity network in a similar manner. Besides, we adopt the Tanimoto coefficient (Hattori *et al.*, 2003) to measure the similarity of drugs based on their chemical structure graph and construct the chemical structure-based drug similarity network.

Meanwhile, FMIDTI also employs the Jaccard similarity coefficient to measure the similarity between targets and establishes the protein-protein interaction-based similarity network, protein-disease association-based network, and drug-protein association-based similarity network respectively. Besides, MIDTI evaluates the similarity value between two targets with the Smith-Waterman score (Smith *et al.*, 1981) based on their primary sequences. The corresponding genome sequences-based similarity network of drugs could be obtained.

## Section 2. Similarity network fusion strategy.

Inspired by BIONIC (Forster *et al.*, 2022), MIDTI will integrate the different similarity networks of drugs and targets with the similarity network fusion strategy. The integrated network could accurately reflect the topologies of the underlying original networks and capture functional information. Different from BIONIC, MIDTI adds a multi-view attention mechanism that adaptively learns the importance of features from different similarity networks.

Here we take the drug similarity networks as an example to demonstrate the fusion process which mainly has four steps. For step one, we feed drug similarity networks into MIDTI, which adopts GCNs to encode different networks separately. The embedding of drugs at $(l+1)$-th layer can be formulated as:

$$H_c^{(l+1)} = \sigma\left(\tilde{D}^{-\frac{1}{2}}\tilde{A}_c\tilde{D}^{-\frac{1}{2}}H_c^{(l)}W_c^{(l)}\right) \tag{2}$$

where $c \in \{1, \ldots, P\}$ is the index number of similarity network and $A_c$ is its adjacency matrix. Besides, $\sigma(\cdot)$ is the ReLU activation function, and $\tilde{A}_c = A_c + I$ is the identity matrix with adaptive size, $\tilde{D}$ is the corresponding degree matrix of $\tilde{A}_c$, $W_c^{(l)}$ is a trainable linear transformation, and $H_c^{(l)} \in \mathbb{R}^{F_m \times M}$ denotes the representations of $M$ drugs that obtained from the $l$-th GCN layer. $F_m$ is the dimension of drug representations.

In step two, MIDTI extracts the embeddings of drugs from $P$ drug similarity networks and applies the multi-view attention mechanism to obtain their discriminative features further. Specifically, Inspired by Hu et al. (Hu *et al.*, 2018), MIDTI firstly adopts the squeeze-and-extract block to assign weights to embeddings of drugs and then utilize CNN to fuse the embeddings of drugs from different similarity networks.

The statistic $z_c$ for $c$-th network is calculated by:

$$z_c = F_{sq}\left(H_c^{(l)}\right) = \frac{1}{F_m \times M}\sum_{i=1}^{F_m}\sum_{j=1}^{M} H_c^{(l)}(i, j) \tag{3}$$

where $H_c^{(l)} \in \mathbb{R}^{F_m \times M}$ is the $c$-th feature matrix for drug. The statistic $Z$ for these $P$ similarity networks is generated by

$$Z = \{z_1, z_2, \ldots, z_P\} \tag{4}$$

MIDTI calculates their view-wise attention (VA) weight for each drug similarity network, which will be formulated as

$$Z_{att} = F_{att}(Z, W_{in}) = \delta(W_2\sigma(W_1 Z)) \tag{5}$$

where $\sigma(\cdot)$ and $\delta(\cdot)$ are Sigmoid and Relu activation function respectively. $W_1$ and $W_2$ are learnable matrices.

Finally, the view-wise attention weight can also be represented as:

$$Z_{att} = \left\{z_{(att,1)}, z_{(att,2)}, \ldots, z_{(att,P)}\right\} \in \mathbb{R}^{F_m \times P} \tag{6}$$

where $z_{(att,c)}$ denotes the learned view-wise attention weight for $c$-th drug similarity network.

Combining the drug features with view-wise attention weight, the drug representation from the

$c$-th network can be defined as:

$$H_{(att,c)} = F_{scale}\left(H_c^{(l)}, z_{(att,c)}\right) = z_{(att,c)} \cdot H_c^{(l)} \tag{7}$$

And MIDTI employs 1D-CNN (Kiranyaz *et al.*, 2021) model to obtain the integrated drug representation matrix $X_d \in \mathbb{R}^{F_m \times M}$.

For step three, MIDTI reconstructs the similarity matrix $S_d$, which is formulated as:

$$S_d = X_d{}^T X_d, S_d \in \mathbb{R}^{M\times M} \tag{8}$$

For step four, MIDTI adopts the sum of mean squared errors as the loss function between the reconstructed matrix and each original drug matrix $\{A_1, A_2, \ldots, A_P\}$. The loss function is defined as follows:

$$L_d = \sum_{c=1}^{P} |A_c - S_d|^2 \tag{9}$$

By minimizing the loss function through back-propagation, MIDTI could establish the integrated drug similarity matrix $A_{homo\_d}$ and the learned drug feature representation $X_d$ after the iteration is finished. Similarly, MIDTI could establish the integrated target similarity matrix and target feature matrix represented as $A_{homo\_t}$ and $X_t$.

### Section 3. Implementation details.

MIDTI is trained based on the Lookahead optimizer with the inner optimizer SGD (Zhang *et al.*, 2019). We adopt the grid search strategy to tune parameters for MIDTI. Specifically, the learning rate is set to 0.1. The number of interactive attention heads is 8. The embedding size of drugs and targets is 512. The numbers of GCN layers, interactive attention layer and MLP layers are equal to 3. During the training process, the dropout value on the deep interactive attention module is 0.1, and the default number of epochs is set to 2000. Furthermore, MIDTI adopts an early stop with a patience of 50.

Besides, we implement our model using a software environment with PyCharm Community Edition 2022.1.1 version and libraries with Python v3.7.0, Numpy v1.21.6, Pytorch v1.10.1, Pandas v0.23.4, Scikit-learn v0.19.2 and Scipy v1.1.0. All experiments are performed on hardware with a desktop computer with one 12th Gen Intel (R) Core (TM) i5-12490F CPU and one NVIDIA GeForce RTX4070 12GB GPU. The detailed implementation information has been published on GitHub (`https://github.com/XuLew/MIDTI`).

### Section 4. Brief introduction for Zheng's and Yamanishi's datasets.

In this study, except for Luo's dataset, we also conduct experiments on Zheng's and Yamanishi's datasets.

Zheng's (Zheng *et al.*, 2018) dataset also contains other drug and target such as chemical structure, drug side effect, drug substitute, and gene ontology. There are 11,819 DTIs, related to 1,094 drugs and 1,556 targets.

Yamanishi's dataset (Yamanishi *et al.*, 2008) contains four sub-datasets, each corresponding to a family of target proteins. They are G Protein-Coupled Receptors (GPCR), Enzymes (Enzyme), Ion Channels (IC) and Nuclear Receptors (NR). The number of known drugs in GPCR, Enzyme, IC, and NR is 223, 445, 210, and 54, respectively, and the number of targets in these classes is 95, 664, 204, and 26, respectively. The number of known drug-target interactions is 635, 2926, 1476 and 90, respectively.

### Section 5. The descriptions for these comparison approaches.

- RF (Pedregosa *et al.*, 2011) is one of the ensemble learning methods for classification and its output is the class selected by most trees. We feed the embeddings of drugs and targets for DTI predictions.
- SVM (Chang and Lin, 2011) is a traditional supervised learning approach and we feed the embeddings of drugs and targets directly to predict the DTIs.
- XGBoost (Chen and Guestrin, 2016) is a powerful machine-learning algorithm that combines the strengths of decision trees and gradient boosting to achieve high predictive accuracy and efficiency.
- GCN (Kipf and Welling, 2016) is a semi-supervised learning approach. Here we feed the drug-target association network into it and learn the embedding of drugs and targets for predicting other potential drug-target pairs.
- GAT (Veličković *et al.*, 2017) is one of the graph neural networks with the attention mechanism. We feed the drug-target association network into GATs and obtain their feature representations for completing the DTI prediction tasks.
- DTI-CNN (Peng *et al.*, 2020) obtains the embeddings of drugs and targets based on the heterogeneous networks and infers their interactions with learned features from a denoising auto-encoder model.

- GCNMDA (Long *et al.*, 2020) builds a heterogeneous network for drugs and microbes and then employs the GCN-based framework with conditional random field (CRF) as well as attention mechanism techniques to discover entity associations. By changing the input to drugs and targets, we can also predict the DTIs.
- MVGCN (Fu *et al.*, 2022) is a model that integrates data through a multi-view graph convolutional network and aims to predict links in a biomedical bipartite network.
- MMGCN (Tang *et al.*, 2021) employs GCN encoder to obtain the embedding of miRNAs and diseases in different similarity views and enhance the learned representations by utilizing a multichannel attention mechanism. We feed the drug similarity network and target similarity network for DTI predictions
- GraphCDA (Dai *et al.*, 2022) is a hybrid graph representation learning framework based on GCN and GAT for predicting disease-associated circRNAs. Here we feed the drug similarity network and target similarity network into this model to learn the features of drugs and targets.
- DTINet (Luo *et al.*, 2017) could learn the low-dimensional vector representation of features, and predict the DTIs with the learned embeddings of drugs and targets via a vector space projection.

## Section 6. Comparison results on Yamanishi's and Zheng's datasets.

In this section, we will compare MIDTI with other baselines on three different datasets, which are (Luo *et al.*, 2017), Yamanishi's (Yamanishi *et al.*, 2008) and Zheng's dataset (Zheng *et al.*, 2018). The results of Luo's dataset have been displayed in the revised manuscript in Section 3.2 and 3.3.

For Yamanishi's dataset, there are four sub-datasets which are GPCR, Enzyme, IC and NR. Here, we also conduct comprehensive evaluation experiments with ACC, AUC and AUPR metrics. Specifically, the results under 1:1, 1:15 and 1:10 ratios have been presented at **Table.S1**, **Table.S2**, **Table.S3**, respectively.

The results of MIDTI and the comparison approach on Zheng's dataset are presented at **Table.S4** respectively. Specifically, on Zheng's dataset, the results under the 1:1 on ACC, AUC and AUPR values are 0.8886, 0.9546 and 0.9497. The results under 1:5 and 1:10 ratios are also shown at the **Table.S4**. On the whole, MIDTI achieves the best performance on this dataset.

The results of this experiment demonstrate that MIDTI has a powerful ability to discover novel interactions between drugs and targets.

## Section 7. Parameter analysis experiments.

In this section, we discuss the sensitivity of several parameters of MIDTI. These parameters mainly include the embedding size, the learning rate, the number of interactive attention heads, the number of GCN layers, the number of interactive attention layers and the number of MLP layers. The corresponding experiment results are all evaluated with ACC, AUC, AUPR, F1 and MCC, respectively. The corresponding results are listed below.

- The embedding size In this experiment, we investigate the effects of embedding size on MIDTI. Here, we set the embedding size as 32, 64, 128, 256 512 and 1024 respectively and the corresponding results are shown in **Fig.S1A**. As the embedding size increases from 32 to 512, the performance of MIDTI has a short decrease at an embedding size of 64, with an overall increasing trend. While its performance decreases with the embedding size increasing from 512 to 1024. The best performance is achieved when the embedding size is 512, and it has values of 0.9340, 0.9787, 0.9701, 0.9370 and 0.8726 on ACC, AUC, AUPR, F1 and MCC, respectively. As a result, we adopt the embedding size of 512 in this study.
- The learning rate

  The results of the Lookahead optimizer with the inner optimizer SGD at different learning rates are shown in **Fig. S1B**. It can be seen that the performance of MIDTI keeps getting better with the increase of the learning rate from 0.01 to 0.1, while its performance gradually decreases when the number of layers becomes larger than 3. When the learning rate is 0.1, MIDTI gets the highest scores, with values of 0.9340, 0.9787, 0.9701, 0.9370 and 0.8726 for ACC, AUC, AUPR, F1 and MCC respectively. Therefore, the learning rate of the model is set to 0.1 in this paper.
- The number of interactive attention heads

  We vary the dimension from 1 to 16 to analyze the performance of MIDTI with different numbers of attention heads. According to the results in **Fig. S1C**, we find that MIDTI with 8 attention heads achieves the best performances. The values on ACC, AUC and AUPR are highest when the number of attention heads is 8, which are 0.9340, 0.9787 and 0.9701, respectively. The scores on F1 and MCC are highest when the number of attention heads is

**Table.S1** The performance of MIDTI as well as other baseline approaches for predicting drug-target interaction at a ratio of 1:1 on Yamanishi's datasets..

| Models | GPCR | | | Enzyme | | | IC | | | NR | | |
|---|---|---|---|---|---|---|---|---|---|---|---|---|
| | ACC | AUC | AUPR | ACC | AUC | AUPR | ACC | AUC | AUPR | ACC | AUC | AUPR |
| RF (Pedregosa *et al.*, 2011) | 0.8142 | 0.8985 | 0.9001 | 0.9023 | 0.9102 | 0.9235 | 0.8872 | 0.9390 | 0.9413 | 0.7056 | 0.8269 | 0.8265 |
| SVM (Chang and Lin, 2011) | 0.7323 | 0.8009 | 0.8534 | 0.8191 | 0.8417 | 0.7854 | 0.7915 | 0.8200 | 0.8199 | 0.6389 | 0.8378 | 0.8196 |
| XGBoost (Chen and Guestrin, 2016) | 0.8118 | 0.8939 | 0.8969 | 0.8992 | 0.9549 | 0.9320 | 0.8916 | 0.9302 | 0.9310 | 0.7667 | 0.8268 | 0.8046 |
| GCN (Kipf and Welling, 2016) | 0.7069 | 0.7159 | 0.7553 | 0.8353 | 0.8648 | 0.8176 | 0.8530 | 0.8628 | 0.8137 | 0.7490 | 0.7588 | 0.7915 |
| GAT (Veličković *et al.*, 2017) | 0.8028 | 0.8197 | 0.8182 | 0.8134 | 0.7751 | 0.7680 | 0.8277 | 0.8556 | 0.8334 | 0.8087 | 0.8471 | 0.8362 |
| DTI-CNN (Peng *et al.*, 2020) | 0.8032 | 0.8335 | 0.8562 | 0.8878 | 0.9243 | 0.9315 | 0.8482 | 0.8917 | 0.8893 | 0.8005 | 0.7337 | 0.7326 |
| GCNMDA (Long *et al.*, 2020) | 0.8281 | 0.8744 | 0.7981 | 0.7883 | 0.5904 | 0.4240 | 0.8027 | 0.9118 | 0.8255 | 0.8201 | 0.8782 | 0.8736 |
| MVGCN (Fu *et al.*, 2022) | 0.8116 | 0.8422 | 0.8255 | 0.8456 | 0.9308 | 0.9153 | 0.8302 | 0.8946 | 0.8862 | 0.8525 | 0.8309 | 0.8346 |
| MMGCN (Tang *et al.*, 2021) | 0.9315 | 0.9361 | 0.9365 | 0.9246 | 0.9448 | 0.9309 | 0.9451 | 0.9376 | 0.9420 | 0.8722 | 0.8556 | 0.8622 |
| GraphCDA(Dai *et al.*, 2022) | 0.8772 | 0.9384 | 0.9413 | 0.9303 | 0.9494 | 0.9325 | 0.9322 | 0.9389 | 0.9305 | 0.7889 | 0.8410 | 0.8364 |
| DTINet (Luo *et al.*, 2017) | 0.8107 | 0.8605 | 0.8321 | 0.8009 | 0.8447 | 0.8819 | 0.7995 | 0.8337 | 0.8732 | 0.7842 | 0.8012 | 0.8008 |
| MIDTI(ours) | **0.9386** | **0.9466** | **0.9448** | **0.9376** | **0.9557** | **0.9441** | **0.9528** | **0.9488** | **0.9502** | **0.8811** | **0.8823** | **0.8776** |

**Table.S2** The performance of MIDTI as well as other baseline approaches for predicting drug-target interaction at a ratio of 1:5 on Yamanishi's datasets..

| Models | GPCR | | | Enzyme | | | IC | | | NR | | |
|---|---|---|---|---|---|---|---|---|---|---|---|---|
| | ACC | AUC | AUPR | ACC | AUC | AUPR | ACC | AUC | AUPR | ACC | AUC | AUPR |
| RF (Pedregosa *et al.*, 2011) | 0.8372 | 0.8617 | 0.8743 | 0.9103 | 0.9336 | 0.9326 | 0.8798 | 0.9271 | 0.9216 | 0.7203 | 0.7972 | 0.8124 |
| SVM (Chang and Lin, 2011) | 0.7490 | 0.7715 | 0.8214 | 0.8328 | 0.8209 | 0.7607 | 0.8071 | 0.8051 | 0.8153 | 0.6701 | 0.8258 | 0.8008 |
| XGBoost (Chen and Guestrin, 2016) | 0.8384 | 0.8766 | 0.8658 | 0.9035 | 0.9406 | 0.9293 | 0.9126 | 0.9247 | 0.9304 | 0.7824 | 0.7948 | 0.7917 |
| GCN (Kipf and Welling, 2016) | 0.8188 | 0.7030 | 0.7476 | 0.8546 | 0.7362 | 0.7417 | 0.8550 | 0.7746 | 0.8035 | 0.7621 | 0.7431 | 0.7838 |
| GAT (Veličković *et al.*, 2017) | 0.8612 | 0.8135 | 0.8067 | 0.8927 | 0.7548 | 0.7494 | 0.8333 | 0.9014 | 0.8639 | 0.8589 | 0.8334 | 0.8104 |
| DTI-CNN (Peng *et al.*, 2020) | 0.8361 | 0.7246 | 0.7231 | 0.8778 | 0.8307 | 0.8380 | 0.8555 | 0.8865 | 0.8716 | 0.8333 | 0.8142 | 0.8148 |
| GCNMDA (Long *et al.*, 2020) | 0.8367 | 0.8548 | 0.7714 | 0.7920 | 0.5593 | 0.3923 | 0.8272 | 0.8824 | 0.8116 | 0.8193 | 0.8938 | 0.8555 |
| MVGCN (Fu *et al.*, 2022) | 0.8460 | 0.8203 | 0.8067 | 0.8622 | 0.9099 | 0.8953 | 0.8302 | 0.8762 | 0.8653 | 0.8521 | 0.8053 | 0.8266 |
| MMGCN (Tang *et al.*, 2021) | 0.9333 | 0.9189 | 0.9023 | 0.9568 | 0.9295 | 0.9156 | 0.9308 | 0.9242 | 0.9203 | 0.8901 | 0.8369 | 0.8319 |
| GraphCDA(Dai *et al.*, 2022) | 0.9280 | 0.9174 | 0.9149 | 0.9566 | 0.9412 | 0.9349 | 0.9331 | 0.9310 | 0.9354 | 0.8815 | 0.8275 | 0.8104 |
| DTINet (Luo *et al.*, 2017) | 0.8254 | 0.8422 | 0.8126 | 0.8241 | 0.8295 | 0.8673 | 0.8103 | 0.8192 | 0.8622 | 0.7902 | 0.8016 | 0.8102 |
| MIDTI(ours) | **0.9418** | **0.9253** | **0.9306** | **0.9677** | **0.9495** | **0.9463** | **0.9414** | **0.9465** | **0.9411** | **0.8921** | **0.9017** | **0.8979** |

1, which are 0.9378 and 0.8744. When using 16 attention heads, the corresponding values are 0.9269, 0.9783, 0.9698, 0.9314 and 0.8615, which is lower by 0.71, 0.04, 0.03, 0.56 and 1.11 than the MIDTI with 8 attention heads. Thus the number of interactive attention heads is set to be 8.

- The number of GCN layers

  To analyze the impact of layer number of GCNs, we vary the number from 1 to 4. The values on ACC, AUC, AUPR, F1 and MCC metrics are lowest when the number of GCN layers is 1, which are 0.9046, 0.9605, 0.9320, 0.9061 and 0.8123. From **Fig.S2A**, we can see that when the number of GCN layers increases to 3, the performance of the model is the best. It has the values of 0.9340, 0.9787, 0.9701, 0.9370 and 0.8726 on ACC, AUC, AUPR, F1 and MCC, respectively. However, the performance of MIDTI decreases with the number of layers increasing from 3 to 4. Thus, the number of GCN layers is set to 3.

**Table.S3** The performance of MIDTI as well as other baseline approaches for predicting drug-target interaction at a ratio of 1:10 on Yamanishi's datasets..

| Models | GPCR | | | Enzyme | | | IC | | | NR | | |
|---|---|---|---|---|---|---|---|---|---|---|---|---|
| | ACC | AUC | AUPR | ACC | AUC | AUPR | ACC | AUC | AUPR | ACC | AUC | AUPR |
| RF (Pedregosa *et al.*, 2011) | 0.8533 | 0.8388 | 0.8486 | 0.9209 | 0.9065 | 0.9015 | 0.8811 | 0.9091 | 0.9125 | 0.7592 | 0.7817 | 0.7977 |
| SVM (Chang and Lin, 2011) | 0.7742 | 0.7581 | 0.8065 | 0.8521 | 0.8075 | 0.7397 | 0.8265 | 0.7861 | 0.7876 | 0.6828 | 0.7958 | 0.7715 |
| XGBoost (Chen and Guestrin, 2016) | 0.8511 | 0.8682 | 0.8359 | 0.9327 | 0.9159 | 0.9142 | 0.9290 | 0.9049 | 0.9101 | 0.7924 | 0.7676 | 0.7738 |
| GCN (Kipf and Welling, 2016) | 0.9012 | 0.6864 | 0.7334 | 0.8735 | 0.7262 | 0.7376 | 0.8804 | 0.7567 | 0.7791 | 0.8083 | 0.7127 | 0.7632 |
| GAT (Veličković *et al.*, 2017) | 0.8721 | 0.7883 | 0.7963 | 0.9001 | 0.7373 | 0.7271 | 0.8369 | 0.8812 | 0.8429 | 0.8911 | 0.8107 | 0.7976 |
| DTI-CNN (Peng *et al.*, 2020) | 0.8449 | 0.6939 | 0.7461 | 0.8805 | 0.8058 | 0.8027 | 0.8625 | 0.8704 | 0.8621 | 0.8633 | 0.7897 | 0.8073 |
| GCNMDA (Long *et al.*, 2020) | 0.8781 | 0.8242 | 0.7593 | 0.8024 | 0.5411 | 0.3702 | 0.8411 | 0.8709 | 0.7857 | 0.8026 | 0.8137 | 0.8393 |
| MVGCN (Fu *et al.*, 2022) | 0.8432 | 0.7993 | 0.7879 | 0.8728 | 0.8908 | 0.8656 | 0.8583 | 0.8508 | 0.8367 | 0.8401 | 0.7838 | 0.8056 |
| MMGCN (Tang *et al.*, 2021) | 0.9303 | 0.9025 | 0.8819 | 0.9661 | 0.9076 | 0.9117 | 0.9462 | 0.9038 | 0.9129 | 0.9025 | 0.8201 | 0.8111 |
| GraphCDA(Dai *et al.*, 2022) | 0.9325 | 0.8874 | 0.9007 | 0.9593 | 0.9240 | 0.9158 | 0.9549 | 0.9117 | 0.9168 | 0.9173 | 0.8053 | 0.7906 |
| DTINet (Luo *et al.*, 2017) | 0.8374 | 0.8261 | 0.7904 | 0.8292 | 0.8103 | 0.8479 | 0.8220 | 0.8007 | 0.8384 | 0.8127 | 0.7729 | 0.7937 |
| MIDTI(ours) | **0.9399** | **0.9088** | **0.9129** | **0.9675** | **0.9375** | **0.9263** | **0.9577** | **0.9205** | **0.9203** | **0.9210** | **0.8354** | **0.8491** |

**Table.S4** The performance of MIDTI as well as other baseline approaches for predicting drug-target interaction under different ratios on Zheng's dataset .

| Models | 1:1 | | | 1:5 | | | 1:10 | | |
|---|---|---|---|---|---|---|---|---|---|
| | ACC | AUC | AUPR | ACC | AUC | AUPR | ACC | AUC | AUPR |
| RF (Pedregosa *et al.*, 2011) | 0.8707 | 0.9489 | 0.9481 | 0.8819 | 0.9136 | 0.8668 | 0.8746 | 0.8935 | 0.8001 |
| SVM (Chang and Lin, 2011) | 0.8547 | 0.9181 | 0.8824 | 0.8522 | 0.9021 | 0.8642 | 0.8492 | 0.9033 | 0.8249 |
| XGBoost (Chen and Guestrin, 2016) | 0.8768 | 0.9440 | 0.9319 | 0.8826 | 0.9293 | 0.8860 | 0.8890 | 0.8909 | 0.8303 |
| GCN (Kipf and Welling, 2016) | 0.8608 | 0.9273 | 0.9100 | 0.8512 | 0.9061 | 0.7818 | 0.8479 | 0.8898 | 0.6926 |
| GAT (Veličković *et al.*, 2017) | 0.8469 | 0.9186 | 0.8933 | 0.8336 | 0.8815 | 0.8668 | 0.8180 | 0.8729 | 0.7527 |
| DTI-CNN (Peng *et al.*, 2020) | 0.8332 | 0.9187 | 0.9132 | 0.8183 | 0.9013 | 0.9003 | 0.8069 | 0.8818 | 0.8817 |
| GCNMDA (Long *et al.*, 2020) | 0.8574 | 0.5405 | 0.3727 | 0.8490 | 0.5163 | 0.3511 | 0.8385 | 0.4963 | 0.3422 |
| MVGCN (Fu *et al.*, 2022) | 0.8267 | 0.9024 | 0.8994 | 0.8183 | 0.8766 | 0.8790 | 0.7947 | 0.8495 | 0.8627 |
| MMGCN (Tang *et al.*, 2021) | 0.8831 | 0.9420 | 0.9425 | 0.8791 | 0.9301 | 0.9159 | 0.8601 | 0.9096 | 0.9056 |
| GraphCDA(Dai *et al.*, 2022) | 0.8167 | 0.6660 | 0.7315 | 0.8314 | 0.5792 | 0.3172 | 0.8939 | 0.6542 | 0.3055 |
| DTINet (Luo *et al.*, 2017) | 0.8472 | 0.9074 | 0.9201 | 0.8325 | 0.8899 | 0.9038 | 0.7989 | 0.8691 | 0.8894 |
| MIDTI(ours) | **0.8886** | **0.9546** | **0.9497** | **0.8984** | **0.9339** | **0.9265** | **0.8986** | **0.9134** | **0.9160** |

Note: The best results are marked in bold and the second best is underlined.

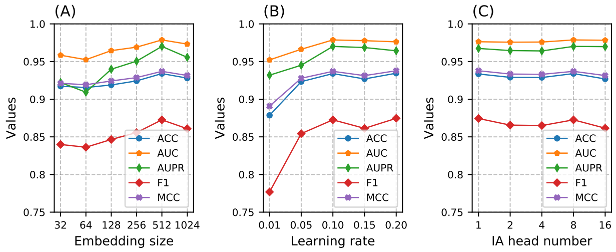


**Fig.S1** The performance of MIDTI under different thresholds for the embedding size, the learning rate and the number of interactive attention heads..

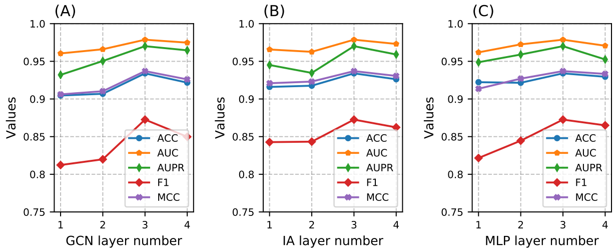


**Fig.S2** The performance of MIDTI under different thresholds for the number of GCN layers, the number of interactive attention layers and the number of MLP layers.

- The number of interactive attention layers

  We investigate the impact of the number of deep interactive attention layers on MIDTI. The number of layers is set to 1, 2, 3 and 4, respectively. The values on n ACC, AUC, AUPR, F1 and MCC, metrics increase when the number of layers is changed from 1 to 3, and then decrease when the number of layers is increased to 4. The results in **Fig.S2B** indicate that MIDTI achieves the best performance when the number of interactive attention layers is 3, its values on five metrics are 0.9340, 0.9787, 0.9701, 0.9370 and 0.8726. In this study, the number of interactive attention layers is set to 3.

- The number of MLP layers

  MLP is employed as the classifier to predict DTIs. It is very critical to choose a proper layer number for MLP. The number of MLP layers is set to 1, 2, 3 and 4. The corresponding results in **Fig.S2C** fully indicate that MIDTI achieves the best performance when the number of MLP layers is 3. It can be seen that the performance of MIDTI keeps getting better with the increase of layer numbers from 1 to 3, while its performance decreases with the increase of layer numbers from 3 to 4. Therefore, the number of MLP layers is set to 3.

## Section 8. Comparison results on different similarity fusion strategies.

In this study, we evaluate the performance of different similarity network fusion strategies, which are MIDTI, MIDTI_ave, and MIDTI_pro respectively. Here, we also select other two similar network fusion strategies, which are called MIDTI_ave and MIDTI_pro. For the MIDTI_ave strategy, we measure the average values from different networks as the integrated similarity values. For MIDTI_pro strategy, the integrated similarity value is formulated as $S = 1 - \prod_{i=1}^{n} S_i$, where $S_i$ denotes the similarity values from the $i$-th similarity network.

The results on Luo's Zheng's and Yamanish's datasets are shown in **Table.S5**. From the results, we could find that the proposed similarity network fusion strategy achieves the best performance on all three datasets, which demonstrates the effectiveness of the MIDTI in finding DTIs.

**Table.S5** The evaluation results of MIDTI with different similarity network fusion strategies on Luo's, Zheng's and Yamanishi's datasets .

| Datasets | Strategy | ACC | AUC | AUPR |
|---|---|---|---|---|
| Luo | MIDTI_ave | 0.9078 | 0.9547 | 0.9336 |
| | MIDTI_pro | 0.8961 | 0.9611 | 0.9501 |
| | MIDTI | **0.9340** | **0.9787** | **0.9701** |
| Zheng | MIDTI_ave | 0.8162 | 0.8789 | 0.8665 |
| | MIDTI_pro | 0.8048 | 0.8803 | 0.8714 |
| | MIDTI | **0.8836** | **0.9546** | **0.9497** |
| Yamanishi_GPCR | MIDTI_ave | 0.8087 | 0.8912 | 0.8676 |
| | MIDTI_pro | 0.7953 | 0.8778 | 0.8440 |
| | MIDTI | **0.9386** | **0.9466** | **0.9448** |
| Yamanishi_Enzyme | MIDTI_ave | 0.8951 | 0.9516 | 0.9387 |
| | MIDTI_pro | 0.8897 | 0.9506 | 0.9358 |
| | MIDTI | **0.9376** | **0.9557** | **0.9441** |
| Yamanishi_IC | MIDTI_ave | 0.8144 | 0.9110 | 0.8935 |
| | MIDTI_pro | 0.8240 | 0.9165 | 0.8982 |
| | MIDTI | **0.9528** | **0.9488** | **0.9502** |
| Yamanishi_NR | MIDTI_ave | 0.7889 | 0.8494 | 0.8332 |
| | MIDTI_pro | 0.7778 | 0.8601 | 0.8409 |
| | MIDTI | **0.8811** | **0.8823** | **0.8776** |

## Section 9. Case study.

In practice, discovering the interactions accurately for some common drugs and targets is another effective manner to verify the effectiveness of DTI prediction models (Tian *et al.*, 2022). In this section, we selected five typical drugs Quetiapine, Clozapine, Aripiprazole, Ziprasidone and Amitriptyline, and analyzed the DTI prediction results of these drugs.

As the same process with previous research (Peng *et al.*, 2020), we exclude all the interactions between the selected drugs and their related targets in the training set and validation set and input the test drugs and their related targets into the test set. In the Amitriptyline set, 23 of 24 known interactions in Luo's dataset are identified. In the Clozapine set, 19 of 20 known interactions are identified. Moreover, all the known interactions are identified in Quetiapine set, Aripiprazole set and Ziprasidone set. These results initially testify that the MIDTI method has a good performance on DTI prediction.

Besides, similar to Xuan's study (Xuan *et al.*, 2022), we list the top 10 targets with predicted scores for the five drugs and verify the predicted results based on DrugBank (Knox *et al.*, 2010), DrugCenter (Avram *et al.*, 2021) and PubChem database (Kim *et al.*, 2023). DrugBank and DrugCentral are web-enabled databases containing comprehensive drug data covering drug function, drug targets and so on. The PubChem database provides up-to-date bioactive drug-like small molecule data and chemical-target interactions, which are abstracted and curated from the primary scientific databases and literature. The corresponding results have been presented in **Table.S6**, which shows the top 10 related targets predicted by MIDTI for the five selected drugs. We can observe that most of the novel DTIs predicted by MIDTI are verified by different databases. For example, Quetiapine is an atypical antipsychotic medication for the treatment of schizophrenia, bipolar disorder, borderline personality disorder, and major depressive disorder (Tandon, 2003). In the top 10 target candidates for Quetiapine, only one target with the gene name NT5C2, is labeled as unproved, meaning that there is no evidence to confirm their interaction. Meanwhile, we also predict the top 10 drugs with the predicted scores for three targets and the results have been displayed in **Table.S7**.

The above analysis indicates that MIDTI has the powerful ability to discover potential drug-target interactions, which has essential implications for drug screening and drug repositioning.

## Section 10. Time and space complexity analysis.

• Time complexity analysis

The time complexity of MIDTI is crucial for its applicability. Here we will analyze its time complexity briefly.

There are mainly three steps for constructing MIDTI, which are presented in Fig.1 in the manuscript. The main task for step one is to construct the multi-type network. Suppose the numbers of drugs and targets are $M$ and $N$. The time complexity for establishing one drug and target similarity network is $O(M^2)$ and $O(N^2)$ respectively. Since MIDTI employs encoders (GCNs) to learn the embeddings of drugs and targets from each similarity network, the time complexity is $O(d_1 \times ME_1F_1)$ and $O(d_2 \times NE_2F_2)$, where $E_1, F_1$ are the number of edges and embedding size from drug similarity networks, $E_2, F_2$ are the number of edges and embedding size form target similarity network, $d_1$ and $d_2$ are the numbers of similarity networks of drugs and targets respectively. For merging these embeddings of drugs and targets, the time complexity is $O(d_1M^2) + O(d_2N^2)$. Besides, the time complexity for constructing the drug-target bipartite network is $O(M \times N)$, and the time complexity for constructing the heterogeneous drug-target network is $O(M^2 + N^2 + MN)$. As a result, the total time complexity for step one is $O(d_1 \times ME_1F_1)+O(d_2 \times NE_2F_2)+O(M^2+N^2+MN)$.

The main task in step two is to feature learning from multi-type networks. MIDTI learns the embedding of drugs from four types

**Table.S6** The top 10 candidate target proteins of five selected drugs.

| Drugname | Rank | UNIPROT ID | Gene Name | Evidence | Rank | UNIPROT ID | Gene Name | Evidence |
|---|---|---|---|---|---|---|---|---|
| | 1 | P08588 | ADRB1 | DrugCentral | 6 | Q13224 | GRIN2B | PubChem |
| Quetiapine | 2 | P28223 | HTR2A | DrugBank | 7 | P08908 | HTR1A | DrugBank |
| DB01224 | 3 | P30939 | HTR1F | DrugCentral | 8 | P49902 | NT5C2 | Unproved |
| | 4 | P31645 | SLC6A4 | PubChem | 9 | P14416 | DRD2 | DrugBank |
| | 5 | P20309 | CHRM3 | DrugBank | 10 | P18089 | ADRA2B | DrugBank |
| | 1 | Q12809 | KCNH2 | DrugCentral | 6 | P25100 | ADRA1D | DrugCentral |
| Clozapine | 2 | P28472 | GABRB3 | PubChem | 7 | Q15822 | CHRNA2 | Unproved |
| DB00363 | 3 | P20309 | CHRM3 | DrugBank | 8 | P08912 | CHRM5 | DrugBank |
| | 4 | Q01959 | SLC6A3 | PubChem | 9 | P31645 | SLC6A4 | DrugCentral |
| | 5 | P41145 | OPRK1 | Unproved | 10 | P08173 | CHRM4 | DrugBank |
| | 1 | P08588 | ADRB1 | DrugBank | 6 | P11388 | TOP2A | Unproved |
| Aripiprazole | 2 | P20309 | CHRM3 | DrugBank | 7 | P28335 | HTR2C | DrugBank |
| DB01238 | 3 | P11387 | TOP1 | Unproved | 8 | P18089 | ADRA2B | DrugBank |
| | 4 | P31645 | SLC6A4 | DrugBank | 9 | P14416 | DRD2 | DrugBank |
| | 5 | P39023 | RPL3 | Unproved | 10 | P41595 | HTR2B | DrugBank |
| | 1 | P31645 | SLC6A4 | DrugCentral | 6 | P28222 | HTR1B | DrugBank |
| Ziprasidone | 2 | P08588 | ADRB1 | DrugCentral | 7 | P30939 | HTR1F | Unproved |
| DB00246 | 3 | P28223 | HTR2A | DrugBank | 8 | P08172 | CHRM2 | DrugBank |
| | 4 | P08908 | HTR1A | DrugBank | 9 | Q13224 | GRIN2B | Unproved |
| | 5 | P23975 | SLC6A2 | DrugCentral | 10 | P11388 | TOP2A | Unproved |
| | 1 | P35372 | OPRM1 | DrugBank | 6 | Q01959 | SLC6A3 | DrugCentral |
| Amitriptyline | 2 | P14867 | GABRA1 | Unproved | 7 | P41143 | OPRD1 | DrugBank |
| DB00321 | 3 | P25021 | HRH2 | DrugBank | 8 | Q15822 | CHRNA2 | Unproved |
| | 4 | P04035 | HMGCR | PubChem | 9 | Q12809 | KCNH2 | DrugBank |
| | 5 | P41145 | OPRK1 | DrugBank | 10 | Q8TCU5 | GRIN3A | Unproved |

**Table.S7** The top 10 candidate drugs of three selected targets.

| Targetname | Rank | UNIPROT ID | Drug Name | Evidence | Rank | UNIPROT ID | Drug Name | Evidence |
|---|---|---|---|---|---|---|---|---|
| | 1 | DB00211 | Midodrine | PubChem | 6 | DB00696 | Ergotamine | PubChem |
| ADRA1A | 2 | DB00334 | Olanzapine | PubChem | 7 | DB00679 | Thioridazine | PubChem |
| P35348 | 3 | DB00449 | Dipivefrin | PubChem | 8 | DB00734 | Risperidone | PubChem |
| | 4 | DB00363 | Clozapine | PubChem | 9 | DB01136 | Carvedilol | PubChem |
| | 5 | DB00450 | Droperidol | PubChem | 10 | DB01186 | Pergolide | PubChem |
| | 1 | DB00334 | Olanzapine | PubChem | 6 | DB01238 | Aripiprazole | PubChem |
| HTR2A | 2 | DB00408 | Loxapine | PubChem | 7 | DB00413 | Pramipexole | Unproved |
| P28223 | 3 | DB00268 | Ropinirole | Unproved | 8 | DB01618 | Molindone | PubChem |
| | 4 | DB00843 | Donepezil | PubChem | 9 | DB06216 | Asenapine | PubChem |
| | 5 | DB01186 | Pergolide | PubChem | 10 | DB01149 | Nefazodone | PubChem |
| | 1 | DB00357 | Aminoglutethimide | PubChem | 6 | DB01406 | Danazol | PubChem |
| CYP19A1 | 2 | DB01217 | Anastrozole | PubChem | 7 | DB00201 | Caffeine | PubChem |
| P11511 | 3 | DB00333 | Methadone | PubChem | 8 | DB00328 | Indomethacin | PubChem |
| | 4 | DB00624 | Testosterone | PubChem | 9 | DB01006 | Letrozole | PubChem |
| | 5 | DB00481 | Raloxifene | PubChem | 10 | DB01234 | Dexamethasone | PubChem |

of networks, and their corresponding time complexity is $O(ME_1F_1)$+ $O(NE_2F_2)$ + $O((M+N)E_3F_3)$+$O((M+N)E_4F_4)$, where $E_3$ and $F_3$ are the number of edges and the embedding size of outputs from the bipartite network, $E_4$ and $F_4$ are the number of edges and the embedding size of outputs from the heterogeneous network.

Step three is to learn the final embeddings of drugs and targets with the deep interactive attention module. Suppose the input embedding size for drugs and targets is $3l$ and the final output embedding size is $F_m$, the time complexity for MHA operation is $O((3l)^2F_m)$, the output of the linear layer is $O((3l)(F_m)^2)$, and the total time complexity for SA/DTA/DTA operation is $O(6l(F_m)^2 + 9l^2F_m)$. The time complexity for running one interactive attention layer is $O(12l(F_m)^2 + 18l^2F_m)$. Moreover, MIDTI performs $(n+l)$interactive attention layer and the time complexity is $O(3l(n+1)^2F_m^2)$. As a result, the total time complexity for step 3 is $O((M+N)(12l+3l(n+1)^2)F_m^2+18l^2F_m)$, where $M$ and $N$ is the number of drugs and targets, $l$ is one constant which denotes the layer number of GCNs in step 2.

In summary, the total time complexity is $O(d_1 \times ME_1F_1)$+$O(d_2 \times NE_2F_2)$+$O(M^2 + N^2+MN)$+ $O(ME_1F_1)$+ $O(NE_2F_2)$ + $O((M+$

$N)E_3F_3)$+$O((M+N)E_4F_4)$ + $O((M+N)(12l+3l(n+1)^2)F_m^2+18l^2F_m)$. Since $d_1$, $d_2$ $F_1$, $F_2$, $F_3$, $F_4$ and $F_m$ are all constant, the total time complexity could be written as $O(ME_1)$+$O(NE_2)$+$O(M^2+N^2+MN)$+ $O(ME_1)$+$O(NE_2)$+$O((M+N)E_3)$+$O((M+N)E_4)$ + $O((M+N)(n+1)^2)$. Since $E_1$ is smaller than $M^2$, $E_2$ is smaller than $N^2$ , and $E_3$, $E_4$ are all smaller than $(M+N)^2$, the total time complexity could be written as $O(M\times M^2+N\times N^2+M^2+N^2+MN)$+ $O(M\times M^2)$+ $O(N\times N^2)$ + $O(2(M+N)^2)$+ $O((M+N)(n+1)^2)\approx O(M^3+N^3+2(M+N)^2+(M+N)(n+1)^2)\approx O(M^3+N^3+(M+N)(n+1)^2)$. Since $n$ is the interactive attention layer, the final time complexity of MIDTI is $O(M^3+N^3)$. The running time for executing one iteration of MIDTI is about 0.5 seconds.

• Space complexity analysis

In this study, there are mainly $M$ drugs and $N$ targets. The sizes of the drug and target similarity matrix are $M^2$ and $N^2$. The size drug-target interaction matrix is $M\times N$. Each drug and target will occupy one Byte of storage space. The interaction relationship will also occupy one Byte of storage space. As a result, the storage space for these networks will be $d_1M^2$ Byte, $d_2N^2$ Byte, $2(MN)^2$ Byte.

Besides, MIDTI utilizes 4 Byte to store the embeddings of drugs and targets, and the corresponding storage space is $2\times4\times(M+N)^2+d_1M^2\times4+d_2\times N^2\times4$. Since MIDTI employs multilayer GCNs to learn the embedding of drugs and targets, the storage space is $l\times(2\times4\times(M+N)^2+d_1\times M^2\times4+d_2\times N^2\times4)$ Byte. As a result, the total space complexity is $(d_1M^2+d_2N^2+2(MN)^2+l(8(M+N)^2+4d_1M^2+4d_2N^2))$B. In practice, the space consumption is about 16 MB.

## References


Avram, S. *et al.* (2021). Drugcentral 2021 supports drug discovery and repositioning. *Nucleic acids research*, **49**(D1), D1160–D1169.

Chang, C.-C. and Lin, C.-J. (2011). Libsvm: a library for support vector machines. *ACM transactions on intelligent systems and technology (TIST)*, **2**(3), 1–27.

Chen, T. and Guestrin, C. (2016). Xgboost: A scalable tree boosting system. In *Proceedings of the 22nd acm sigkdd international conference on knowledge discovery and data mining*, pages 785–794.

Dai, Q. *et al.* (2022). Graphcda: a hybrid graph representation learning framework based on gcn and gat for predicting disease-associated circrnas. *Briefings in Bioinformatics*, **23**(5), bbac379.

Forster, D. T. *et al.* (2022). Bionic: biological network integration using convolutions. *Nature Methods*, **19**(10), 1250–1261.

Fu, H. *et al.* (2022). Mvgcn: data integration through multi-view graph convolutional network for predicting links in biomedical bipartite networks. *Bioinformatics*, **38**(2), 426–434.

Hattori, M. *et al.* (2003). Development of a chemical structure comparison method for integrated analysis of chemical and genomic information in the metabolic pathways. *Journal of the American Chemical Society*, **125**(39), 11853–11865.

Hu, J. *et al.* (2018). Squeeze-and-excitation networks. In *Proceedings of the IEEE conference on computer vision and pattern recognition*, pages 7132–7141.

Kim, S. *et al.* (2023). Pubchem 2023 update. *Nucleic acids research*, **51**(D1), D1373–D1380.

Kipf, T. N. and Welling, M. (2016). Semi-supervised classification with graph convolutional networks. *arXiv preprint arXiv:1609.02907*.

Kiranyaz, S. *et al.* (2021). 1d convolutional neural networks and applications: A survey. *Mechanical systems and signal processing*, **151**, 107398.

Knox, C. *et al.* (2010). Drugbank 3.0: a comprehensive resource for omics research on drugs. *Nucleic acids research*, **39**(suppl_1), D1035–D1041.

Long, Y. *et al.* (2020). Predicting human microbe–drug associations via graph convolutional network with conditional random field. *Bioinformatics*, **36**(19), 4918–4927.

Luo, Y. *et al.* (2017). A network integration approach for drug-target interaction prediction and computational drug repositioning from heterogeneous information. *Nature communications*, **8**(1), 573.

Niwattanakul, S. *et al.* (2013). Using of jaccard coefficient for keywords similarity. In *Proceedings of the international multiconference of engineers and computer scientists*, volume 1, pages 380–384.

Pedregosa, F. *et al.* (2011). Scikit-learn: Machine learning in python. *the Journal of machine Learning research*, **12**, 2825–2830.

Peng, J. *et al.* (2020). A learning-based method for drug-target interaction prediction based on feature representation learning and deep neural network. *BMC bioinformatics*, **21**(Suppl 13), 394.

Smith, T. F. *et al.* (1981). Identification of common molecular subsequences. *Journal of molecular biology*, **147**(1), 195–197.

Tandon, R. (2003). Improvement without impairment: a review of clinical data for quetiapine in the treatment of schizophrenia. *Journal of clinical psychopharmacology*, **23**(3), S15–S20.

Tang, X. *et al.* (2021). Multi-view multichannel attention graph convolutional network for mirna–disease association prediction. *Briefings in Bioinformatics*, **22**(6), bbab174.

Tian, Z. *et al.* (2022). Mhadti: predicting drug–target interactions via multiview heterogeneous information network embedding with hierarchical attention mechanisms. *Briefings in Bioinformatics*, **23**(6), bbac434.

Veličković, P. *et al.* (2017). Graph attention networks. *arXiv preprint arXiv:1710.10903*.

Xuan, P. *et al.* (2022). Gvdti: graph convolutional and variational autoencoders with attribute-level attention for drug–protein interaction prediction. *Briefings in bioinformatics*, **23**(1), bbab453.

Yamanishi, Y. *et al.* (2008). Prediction of drug–target interaction networks from the integration of chemical and genomic spaces. *Bioinformatics*, **24**(13), i232–i240.

Zhang, M. *et al.* (2019). Lookahead optimizer: k steps forward, 1 step back. *Advances in neural information processing systems*, **32**.

Zheng, Y. *et al.* (2018). Predicting drug targets from heterogeneous spaces using anchor graph hashing and ensemble learning. In *2018 International Joint Conference on Neural Networks (IJCNN)*, pages 1–7. IEEE.
